# Modeling the Transmission of the SARS-CoV-2 Delta Variant in a Partially Vaccinated Population

**DOI:** 10.3390/v14010158

**Published:** 2022-01-16

**Authors:** Ugo Avila-Ponce de León, Eric Avila-Vales, Kuanlin Huang

**Affiliations:** 1 Programa de Doctorado en Ciencias Biológicas, Universidad Nacional Autónoma de México, Mexico City 04510, Mexico; ugo.avila.ponce@gmail.com; 2 Facultad de Matemáticas, Universidad Autónoma de Yucatán, Anillo Periférico Norte, Tablaje Catastral 13615, Mérida 97119, Mexico; 3 Center for Transformative Disease Modeling, Department of Genetics and Genomic Sciences, Tisch Cancer Institute, Icahn Institute for Data Science and Genomic Technology, Icahn School of Medicine at Mount Sinai, New York, NY 10029, USA

**Keywords:** vaccines dynamics, mathematical model, SARS-CoV-2 variants, sensitivity analysis: control reproduction number, breakthrough cases

## Abstract

In a population with ongoing vaccination, the trajectory of a pandemic is determined by how the virus spreads in unvaccinated and vaccinated individuals that exhibit distinct transmission dynamics based on different levels of natural and vaccine-induced immunity. We developed a mathematical model that considers both subpopulations and immunity parameters, including vaccination rates, vaccine effectiveness, and a gradual loss of protection. The model forecasted the spread of the SARS-CoV-2 delta variant in the US under varied transmission and vaccination rates. We further obtained the control reproduction number and conducted sensitivity analyses to determine how each parameter may affect virus transmission. Although our model has several limitations, the number of infected individuals was shown to be a magnitude greater (~10×) in the unvaccinated subpopulation compared to the vaccinated subpopulation. Our results show that a combination of strengthening vaccine-induced immunity and preventative behavioral measures like face mask-wearing and contact tracing will likely be required to deaccelerate the spread of infectious SARS-CoV-2 variants.

## 1. Introduction

Severe acute respiratory syndrome coronavirus 2 (SARS-CoV-2) was first reported in late 2019 and has since amounted to over 275 million confirmed cases of COVID-19 around the globe at the time of writing [1]. Throughout 2020, most countries relied on applying nonpharmaceutical interventions (NPIs), such as social distancing, face masks, and partial or total lockdown, among others, to contain the spread of SARS-CoV-2. Nevertheless, these measures were insufficient in most countries to mitigate the COVID-19 pandemic [2,3]. Multiple mathematical models have been developed to evaluate the spread of the virus under different behavior patterns and NPI usage [4], showing how NPI, including the use of face-masks [5,6,7], can significantly mitigate the spread of the virus [8]. However, social distancing for a prolonged period can affect individuals’ mental health [9]. At the end of 2020, the first SARS-CoV-2 vaccines were approved for emergency use [10,11]. The dynamics of COVID-19 spreading are directly affected by the vaccination roll-out and the emergence of new SARS-CoV-2 variants showing varied rates of transmission and resistance [12]. To better forecast the future trends of the COVID-19 pandemic [13], new mathematical models are needed to fully factor in the vaccination roll-out and the interactions of different variant strains with the vaccines [14]. 

SARS-CoV-2 is an RNA virus capable of mutagenesis, and multiple new variants have emerged throughout the ongoing COVID-19 pandemic. Based on their varied levels of infectiousness, lethality, and response to the vaccine, the most threatening ones are identified as variants of concern (VOCs) [12]. Succeeding the alpha variant (B.1.1.7 under the Pango lineage) that gained prevalence in the first half of 2021, the delta variant (B.1.617.2, AY.1, AY.2, and AY.3) has become the most prominent strain among the sequenced variants in many countries by September 2021 [15,16]. While most of the approved vaccines still demonstrate effectiveness against SARS-CoV-2, the delta variant’s enhanced transmission rate and resistance to vaccines [17] imply that the trajectory of the COVID-19 pandemic will depend on several parameters associated with vaccine-induced immunity against new variants.

Vaccination is an effective strategy to control the spread of infectious diseases and has even eradicated diseases such as smallpox [18]. In the US, mass vaccination began on December 14th, 2020 with BNT162b2 (Pfizer/BioNTech, two doses), followed by mRNA-1273 (Moderna; two doses) and Ad26.COV2.S (Janssen J&J, one dose). By the beginning of November 2021, 58.2% of the US population had been fully vaccinated by either of these three vaccines applied in the US [19,20]. While vaccines deployed in the US and the world are highly effective in controlling the spread of the original SARS-CoV-2 strains, they show different effectiveness against new strains like the delta variant [21]. Previous studies have derived the fraction of vaccinated individuals required to achieve herd immunity [22], but these models do not consider the real-world vaccine effectiveness against new strains. In this paper, we propose a differential equations model to simulate the spread of COVID-19 in a partially vaccinated population (i.e., the US population towards the end of 2021), showing the distinct dynamics in the unvaccinated and vaccinated subpopulations under scenarios of varied transmission rates and vaccine effectiveness.

## 2. Materials and Methods

### 2.1. Overview

In the following subsections, we describe the derivation of the mathematical formulas and parameter estimation for the model, including the parameters to explain the behavior of an imperfect vaccine. We also document the procedures for obtaining the control reproduction numbers, as well as the local and global sensitivity analyses.

### 2.2. Derivation of the Mathematical Model of a Partially-Vaccinated Population of the United States

**Figure 1:**
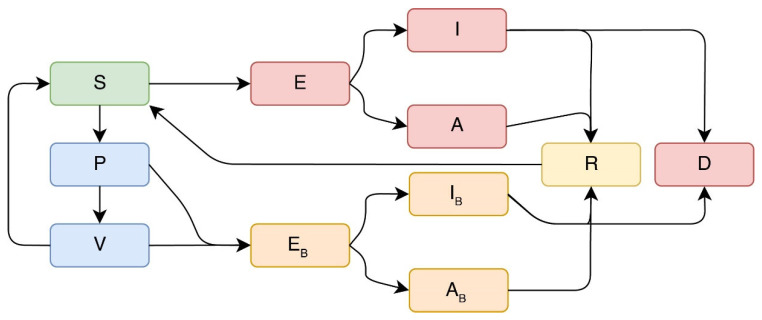
Schematic of the model considering vaccinated vs. unvaccinated individuals and the projection of newly infected symptomatic and asymptomatic cases. Flow dynamics of the mathematical model. S: susceptible, P: partial immunity (one vaccine dose), and V: full immunity (two vaccine doses). E: unvaccinated, exposed. I: unvaccinated, infected, and symptomatic, A: unvaccinated, infected, and asymptomatic. $E_{B}$: vaccinated, exposed.$\;I_{B}$: vaccinated, infected, and symptomatic (“breakthrough cases”). $A_{B}$: vaccinated, infected, and asymptomatic (“breakthrough cases”). R: recovered. D: deceased.

We modified a previously developed compartmental model [23], where the vaccination was incorporated in a model of COVID-19 in Mexico. The adaptation was integrated with Reference [24] to accurately model the impact of an imperfect vaccine in a homogenous model. The mathematical model contains two separate sets of equations for the delta variants that have the ability to affect susceptible populations and vaccinated populations regardless of the number of vaccine doses. Our SPFEIARD model evaluates the dynamics of ten populations at any given time t, which are denoted as S(t), E(t), etc. An illustrative representation of the flow through the subpopulations is depicted in Figure 1. Our mathematical model does not include any natural recruitment (births) or any death not related to COVID-19. Susceptible individuals will decrease after an infection, a characteristic acquired because of an interaction with a symptomatic infected individual at a rate $\beta_{1}$ or asymptomatic individuals at a rate $\beta_{2}$. Once exposed to the virus (E(t)), the infected individuals will become infectiousness at a rate of 1/w days. Among these individuals, only a proportion will develop symptoms at a rate $p_{1}$, whereas the rest of the exposed individuals will be asymptomatic. The symptomatic subpopulation incur death from COVID-19 at a rate $\delta_{1}$. Asymptomatic and symptomatic infected individuals will recover at a rate γ.

The second set of equations acknowledge the effects of vaccination and immunity. Susceptible individuals will be vaccinated at a rate ρ(t)≥0. After receiving the first dose, individuals will enter the partial-immunity compartment and flow to the full-immunity compartment once they receive the second dose at a rate 1/λ days. Partial and full-immunity individuals are considered susceptible at a reduced risk due to the imperfect vaccine protection against the delta variant [25,26]. Therefore, vaccinated individuals can be exposed $\left(E_{B}\right)$ and infected $\left(I_{B},A_{B}\right)$, and such infections can also be referred to as “breakthrough infections”. Individuals flow from partial immunity to infected at a rate of $\left(1-\varepsilon_{L1}\right)*\beta_{1}$ after interactions with a symptomatic individual and a rate of $\left(1-\varepsilon_{LA1}\right)*\beta_{2}$ if they interact with an asymptomatic individual. Similarly, individuals with full immunity can get infected by a symptomatic or asymptomatic individual at a rate of $\left(1-\varepsilon_{L2}\right)*\beta_{1}$ or $\left(1-\varepsilon_{LA2}\right)*\beta_{2}$, respectively. Breakthrough cases become infectiousness at a rate of 1/w days and become symptomatic at a rate $p_{2}$, which is different from non-vaccinated individuals due to the ability of vaccine to reduce symptoms. Finally vaccinated symptomatc or asymptomatic individuals decease at a rate of $\delta_{2}$, which is lower than $\delta_{1},$ due to the protection provided by a vaccine. A full description of the model and the set of equations is in Appendix A.1, and further description of the parameters is in Table S1.

### 2.3. Estimation of Parameters Related to an Imperfect Vaccine

We simulated immunity acquired by one dose or two doses of the BNT162b2 vaccine (the first FDA-approved COVID-19 vaccine) against the delta variant, because it is the vaccine most widely applied in the US (~60% of US vaccinated individuals have received BNT162b2 [19]) and the availability of real-world effectiveness data [27,28] in the period during which the delta strain dominated. Vaccine effectiveness or leakiness occurs when the vaccine reduces but does not eliminate the risk for infection. Vaccine effectiveness may change over time as new variants carrying different mutations emerge. The effectiveness data first published in December of 2020 likely related to strains similar to the original virus that emerged from Wuhan [29], and as new strains emerge (i.e., alpha, delta, and omicron), the vaccine effectivity may reduce. We thus obtained real-world data from a study analyzing data from around 13 days (between 24 June and 12 July 2021) of the REal-time Assessment of Community Transmission-1 (REACT-1) study in the UK [27], during which the delta variant was the dominating strain. In REACT-1, the vaccine effectiveness was obtained by comparing odds ratios between vaccinated and unvaccinated individuals using a logistic regression adjusted for age, sex, region, ethnicity, and index of multiple deprivation (IMD) and was separately determined for one or two dose against the risk of a symptomatic or asymptomatic infection. Based on this real-world data, vaccine effectiveness for the delta variant for only one dose for symptomatic COVID-19 is 0.35 (95% confidence interval (CI), 0.22–0.4). For two doses, the effectiveness is 0.59 (CI, 0.5–0.78). For asymptomatic infections, the effectiveness for one dose is 0.3 (CI, 0.22–0.4) and, for two doses, is 0.49 (0.45–0.64). We also included all-or-nothing protection, which means people who received the vaccine but the vaccine fails to protect a $\varepsilon_{a}$ fraction of individuals. This value is 0.0862 (CI 0.0689–0.10344). Another important parameter is the relative transmission. A vaccine may fail to protect an individual from getting infected, but it can still reduce the effect of the presence of symptoms and infectiousness. We denote this parameter as $\mu_{1}$ (0.94, CI 0.84–1.05) and $\mu_{2}$ (0.73, CI 0.59–0.9) for the relative transmission with one dose or two doses, respectively [30]. Finally, waning immunity describes that protection decreases over time, and vaccinated individuals become fully susceptible to infection at a rate α. Parameter α was assumed herein by vaccine-induced immunity being worn off after six months and represented a conservative estimate that may apply for a variant with high resistance to vaccine-induced immunity. We also obtained the transmission rates for symptomatic and asymptomatic infections by fitting the parameters using the data of daily infections and deaths provided by the repository developed by the Johns Hopkins University [1].

### 2.4. Estimation of the Function to Predict Vaccination in the following Months

Given that, in real-world situations, the vaccination rate is rarely a linear function defined by a constant daily dose, our model incorporates a piecewise function of the vaccination rate based on the applied daily doses from 20 December 2020 to 31 October 2021 [18]. Different ranges for the vaccination rate are shown in Table S2. The value of ρ(t) is the average value of the doses applied in the interval of days described in Table S2.

### 2.5. Estimation of the Parameters of the Outbreak of the Delta Virus in the US

To describe the evolution of the ongoing COVID-19 pandemic in the US, taking into account the vaccination rate and the level of implemented NPI, we assume that the infection, recovery rate, and death rate are time-dependent functions like described in References [23,31,32].

To model the death rate $\delta_{1}$, we assume that it will decrease with time due to an increase of more vaccinated individuals or the development of more advanced treatments. Therefore, we can model $\delta_{1}$ with the following equation:

$$\delta_{1}\left(t\right)=\delta_{0}exp\left(-\frac{t}{t_{\delta }}\right)+\delta_{1},$$

where $\delta_{0}+\delta_{1}$ is the initial death rate, and it will decrease at a characteristic time $t_{\delta }$ until it reaches the value of $\delta_{1}$. 

The recovery rate may also vary over time due to medical improvements in therapeutics or immunity provided by a vaccine. Hence, we model the recovery rate using the following function:

$$\gamma \left(t\right)=\gamma_{0}+\frac{\gamma_{1}}{1+\exp\left(-t+t_{\gamma }\right)},$$

where $\gamma_{0}$ is the rate of recovery at time 0, and $\gamma_{0}+\gamma_{1}$ the eventual recovery rate that will be reached at the time $\tau_{\gamma }$.

Finally, to model the effect of the pandemic measures (application of vaccines), which causes the infection rate to decrease over time, this rate can be modeled by the following function:

$$\beta_{1}\left(t\right)=\beta_{0}exp\left(-\frac{t}{t_{\beta }}\right)+\beta_{1},$$

where $\beta_{0}+\beta_{1}$ is the initial infection rate that will decrease at a characteristic time $t_{\beta }$ until it reaches the value of $\beta_{1}$.

The model also included two fixed parameters obtained from the literature and the WHO COVID-19 report listed in Table S1, including the average length of the latent period (w) and the proportion of symptomatic individuals $\left(p_{1}\right).$ The system of differential equations was solved using Matlab 2016b with the ode45 solver, which is based on the explicit Runge–Kutta formula. Our model was calibrated using the data of COVID-19 infections and deaths from the period of June 2021 to 31 October 2021, during which the delta variant was the dominant strain throughout the US [20]. The data was obtained from the open-source repository of the Johns Hopkins University repository [1], and our code and implementation of the model can be downloaded at https://github.com/UgoAvila/Delta-Variant-In-the-US (last accessed on 23 December 2021).

The optimization of the parameters was performed in two steps: The first step was to minimize the Sum of Squared Errors (*SSE*), where, for a given vector of parameters x, we compute the numerical solutions for our system, and the cumulative number of infected cases with symptoms, C(t)≔I+RI+D, where *I* is infected, *R* is recovered, and *D* is deceased; we also compute the cumulative number of infected cases without symptoms, A(t)≔A+RA.

The Sum of Squared Errors is given by:

$$SSE\left(x\right)=\overset{n}{\underset{i=1}{\sum }}\left[k_{1}{\left(C\left(t_{i}\right)-C_{i}^{exp}\right)}^{2}+k_{2}{\left(D\left(t_{i}\right)-D_{i}^{exp}\right)}^{2}+k_{3}{\left(A\left(t_{i}\right)-A_{i}^{exp}\right)}^{2}\right],\;$$

where $C_{i}^{exp},\;D_{i}^{exp}$, and $A_{i}^{exp}$ denote the experimental data for cumulative infections with the symptoms, deaths, and infections asymptomatic reported for day $t_{i}\left(i=1,2,\ldots .n\right),$ and $k_{1},\;k_{2},$ and $k_{3}$ are coeffiecients used to compensate the order of magnitude of the data. We used $k_{1}=20$, $k_{2}=10,$ and $k_{3}=1$. We applied three searches to minimize the SSE function: first, a gradient-based model; next, a gradient-free algorithm; and finally, another a gradient-based method for a precise approximation.

In the second step, we used the set of parameters obtained by minimizing the SSE as the initial value of a Markov Chain Monte Carlo (MCMC) approach, where we set the iteration number to 8000 and the first 6000 iterations as the burn-in period. We computed the solution of the model for each set of parameters obtained after the burn-in period and further calculated the mean and standard deviation for these solutions. The stabilized estimates of the death, recovery, and infection rates are listed in Table S1, and how our model fits with the data of infection and death is depicted in Figure S1.

### 2.6. Estimation of the Control Reproduction Number 

In our mathematical model, there exists a disease-free equilibrium that happens when the vaccination rate ρ=0. Additionally $E=I=A=E_{B}=I_{B}=A_{B}=0$, and the disease-free equilibrium is:

$$x_{DF}=\left(S^{*},0,0,0,0,V^{*},0,0,0,R^{*},0\right).$$

where $S^{*}\geq 0$, $R^{*}>0,$ and $V^{*}>0$. 

This equilibrium represents where the vaccination roll-out has ended, resulting in fixed fractions of the vaccinated and susceptible populations. We compute the control reproduction number $R_{c}$ in this disease-free equilibrium by applying the next-generation method to find $R_{c}$ and solving the following equation: $R_{c}=\rho \left(FV^{-1}\right)$^1^, where *F* is the derivatives of the new infections, *V* is the transition matrix (flow between compartments), and ρ is the spectral radius.

### 2.7. Local Sensitivity Analysis of the Parameters of the Mathematical Model

The sensitivity analysis of the control reproduction number was carried out using the following definition [33]: If $R_{c}$ is differentiable with respect to a given parameter θ, then the normalized forward sensitivity index of $R_{c}$ is defined by:

$$\Gamma_{\theta }^{R_{c}}=\frac{\theta }{R_{c}}*\frac{\partial R_{c}}{\partial \theta }.$$

Once we obtained the parameters, we were interested in perturbing them to determine how their changes affect the reproduction number, and we solved the equations using Mathematica. After we had the partial derivation of a parameter, we fixed the value of the other parameters fitted from data or obtained from other studies and computed the index of the parameters. A positive sign of the parameter correlated with an increase of the control reproduction, whereas a negative sign is associated with a decrease of the control reproduction number. 

### 2.8. Global Sensitivity Analysis of the Mathematical Model

In this subsection, we applied and adapted the global sensitivity analysis approach described in Reference [34] to our model. We sampled the 17 included parameters and evaluated which were important in determining the behavior of our model. We applied the analysis by using partial rank correlation coefficient (PRCC) by sampling using the Latin hypercube method. These methods allow us to evaluate which parameter affects our response function. In our case, we used the eleven differential equations as our response function. The PRCC values range between −1 and 1, where a negative value means that parameter is negatively correlated with the response function evaluated, and a positive value suggests a positive correlation between the parameter with the response function be evaluated. The significance of each correlation was evaluated, and we only reported parameters whose correlations with the response function showed a *p*-value ≤0.05. 

## 3. Results

### 3.1. Modeling the SARS-CoV-2 Delta Variant Spread in a Partially Vaccinated Population

In real-world situations, the vaccination rate is rarely a linear function defined by a constant daily dose. Thus, our model incorporates a piecewise function of vaccination rate ρ(t) based on the applied daily doses from 20 December 2020 to 31 October 2021 [20]. The projection of this baseline vaccination rate (VR) estimates that the US, by the end of August 2021, contained ~60% of the population with one dose (Figure S2A), and ~59% individuals with two doses of the vaccine (Figure S2B) and, by January, ~60% with one dose and ~50% with two doses (Figure S1C,D). The real-world VR may vary due to a wide range of factors, and thus, we simulated different VRs from mid-November of 2021 based on this VR function in subsequent applications. 

### 3.2. Spread of the Delta Variant under Different Transmission and Vaccination Rates

**Figure 2:**
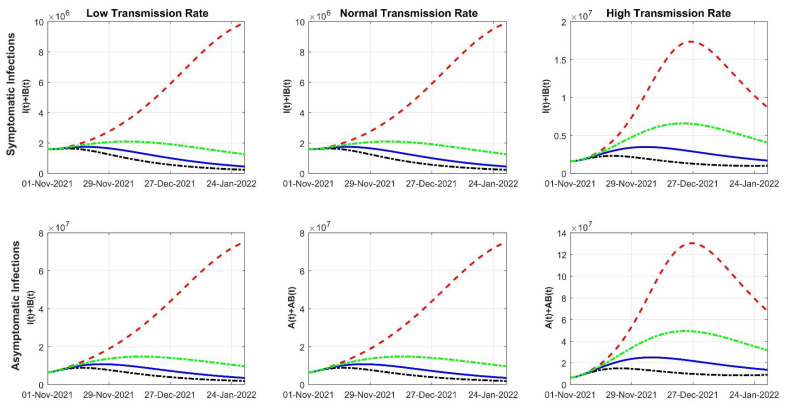
Modeled dynamics of the new infections that are symptomatic (top row) and asymptomatic (bottom row) from the SARS-CoV-2 delta variant considering different vaccination rates. The red line presents zero vaccination, the green line represents a 50% decrease in VR, the blue line means baseline VR, and the black dotted line denotes 200% VR.

Using this model, we evaluated how different VRs might affect the spread of the SARS-CoV-2 delta variant. We further considered low, normal, and high transmission rates, which could reflect different implementation levels of nonpharmaceutical strategies (NPI). Figure 2 shows the projections of new SARS-CoV-2 symptomatic and asymptomatic infections, which included the modeled solutions assuming no vaccination (red dotted line), 50% decrease of VR (green dotted line), baseline VR (blue solid line), and 200% VR (black dotted line). Regardless of the transmission rate, the case counts would rise exponentially in a hypothetical US population with zero vaccination. Given a low transmission rate, new infections would plateau to 2.00 × 10^6^ symptomatic cases per day given 50% VR until the end of December 2021. Given a baseline VR, symptomatic cases would plateau in the third week of November at a value of 1.85 × 10^6^. For 200% VR, symptomatic cases would reach a value of 1.62 × 10^6^ before tapering down between November and December of 2021. The forecast for asymptomatic individuals would behave in a similar manner as symptomatic individuals (Figure 2). Under a normal transmission rate, a US population with 50% VR would rise to a peak of 2.0 × 10^6^ symptomatic cases and 1.85 × 10^7^ asymptomatic cases per day. In comparison, the baseline and 200% VR population would have a more moderate pandemic, peaking at 2.0 × 10^6^ and 1.86 × 10^6^ symptomatic cases per day, respectively (Figure 2). Under a high transmission rate, the new infection counts would be significantly higher even under a baseline VR, reaching a peak of up to 0.45 × 10^7^ symptomatic cases and 2.2 × 10^7^ asymptomatic cases per day. A 200% VR would best control the pandemic in this high transmission rate population, where the peak of new daily infections would still rise to 0.25 × 10^7^ symptomatic cases and 1.85 × 10^7^ asymptomatic cases (Figure 2). Under 200% VR, the cases would start to decrease between November and December 2021, and this trend would continue through January 2022 with daily infections of approximately 0.1 × 10^7^ symptomatic cases and 1.00 × 10^7^ asymptomatic cases. Regardless of the transmission rate, there would be a much higher number of individuals recovered from the disease mostly due to more infections (Figure S3A,C,E). There is a slight difference in recovered dynamics based on the different VRs (Figure S3A,C,E). Compared to a 50% decrease of the baseline VR, a higher VR is projected to reduce roughly 250,000 deaths if the US maintains the baseline VR and 300,000 deaths if the VR is accelerated to 200% (Figure S3B,D,F).

### 3.3. Vaccine Effectiveness and New Infections in Vaccinated vs. Unvaccinated Individuals

Vaccinated individuals have consistently shown a significantly lower rate of contracting COVID-19 and a reduced ability to transmit the virus. However, since the emergence of SARS-CoV-2 variants capable of immune evasion, an elevated number of breakthrough cases have been reported. We dissected the modeled results to determine new infections that would arise from vaccinated vs. unvaccinated subpopulations. In addition to using the estimate of vaccine effectiveness, we also constructed models of low and high vaccine effectiveness based on the upper and lower bound of the 95% confidence intervals. Low vaccine effectiveness is modeled as 0.22 for one-dose vaccinated individuals for either symptomatic and asymptomatic infections and, for fully vaccinated individuals, 0.5 for asymptomatic and 0.45 for asymptomatic infections. High vaccine effectiveness is modeled as 0.4 for one-dose vaccinated individuals for either symptomatic and asymptomatic infections and, for fully vaccinated individuals, 0.78 for symptomatic and 0.64 for asymptomatic infections.

**Figure 3:**
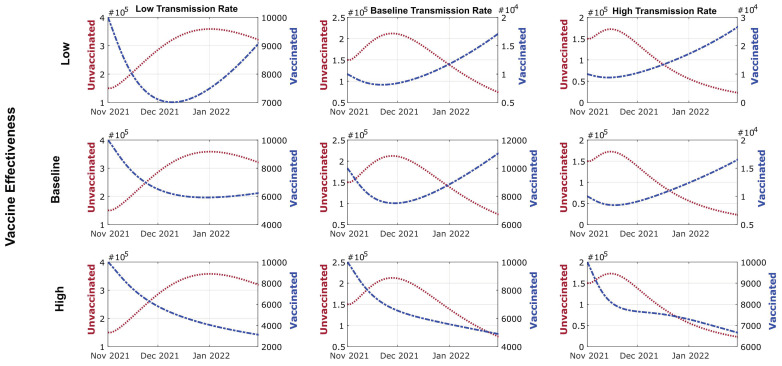
Modeled projections of symptomatic infections in unvaccinated and vaccinated subpopulations under different vaccination rates and vaccine effectiveness. The case counts of the unvaccinated individuals are depicted by the red line and the unit labels on the left-side *y*-axis, whereas the infected, vaccinated (breakthrough) cases are depicted by the blue line and the unit labels on the right-side *y*-axis.

The models showed that, even after accounting for the reduced vaccine effectiveness against the delta variant, the number of symptomatic infections contributed by the unvaccinated individuals is generally an order of magnitude (~10×) higher than that from the vaccinated individuals under all scenarios (Figure 3). Given 50% VR, new symptomatic infections will continue to rise in unvaccinated individuals and surpass 1.0 × 10^4^ cases by the end of January 2022, regardless of vaccine effectiveness. Considering 50% VR in a scenario of low or baseline transmission, breakthrough cases will decrease at the end of 2021 but increase through January 2022, when an even greater fraction of the population becomes vaccinated and infected (Figure S4). Only in the case of high transmission (no or minimal NPI) will breakthrough cases start decreasing early in November 2021 in contrast with unvaccinated individuals until early January 2022, when breakthrough cases will increase with unvaccinated individuals (Figure S4). Given the baseline VR and varying transmission rates, unvaccinated infected individuals will continue to increase until the number peaks mid-December 2021 with 2.5 × 10^5^. Meanwhile, breakthrough cases will decrease in the following months and start increasing by January 2022. Given the high vaccine effectiveness, breakthrough and unvaccinated cases will decrease earlier under a baseline transmission rate (Figure S5). Under a high transmission rate, breakthrough cases will increase in the following months, while unvaccinated cases will decrease to the plateau in January 2021 (Figure S6). Given 200% VR, symptomatic infections of unvaccinated individuals will peak at the end of November 2021 and then decrease into January 2021. At 200% VR, breakthrough cases and nonvaccinated individuals will show parallel trends in these months (Figure S6). Notably, due to the higher number of individuals becoming vaccinated over time under the baseline or 200% VR scenarios, new symptomatic infections from vaccinated individuals (“breakthrough cases”) were projected to rise in December 2021 regardless of vaccine effectiveness. Asymptomatic infections arising in the next four months showed a similar trend compared to symptomatic infections across different VR and vaccination effectiveness, albeit at a higher magnitude (Figure S7). Different vaccination rates would also alter the trajectory of cumulative COVID-19-related deaths, where more deaths could be prevented given a higher VR. For example, 200% VR may have 125,000 less deaths compared with 50% VR and ~50,000 less deaths when compared with the baseline VR. If the US maintains the baseline VR, it may have ~100,000 less compared with 50% VR (Figure S8). While the effect of altering the vaccine effectiveness and rates may be predicted without complex modeling, our model dissects the new case and death counts arising in vaccinated vs. unvaccinated subpopulations and may be useful in formulating public health decisions. 

### 3.4. The Control Reproduction Number Mediated by Natural and Vaccine-Induced Immunity

**Figure 4:**
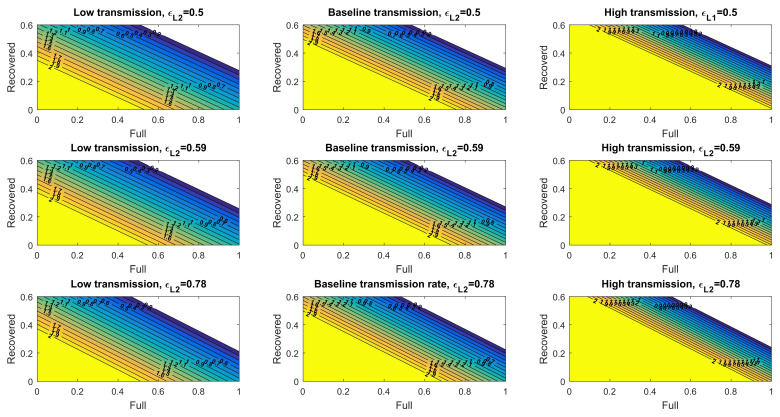
Control reproduction number for symptomatic transmission considering full immunity and recovered individuals.

A pandemic declines when the control reproduction number ($R_{c}$)—the average number of new infections generated by an infected individual over the infected period in a controlled population (i.e., one with a vaccination programs)—is lower than one ($R_{c}<1$). A disease-free equilibrium may be achieved when sufficient fractions of the population are fully vaccinated and recovered (natural immunity) individuals. We derived $R_{c}$ and obtained its value under different levels of transmission rates and vaccine effectiveness (supplementary methods). Under a baseline transmission rate, and given that 30% of the population recovered from the disease and acquired natural immunity, over 69%, 67%, and 62% of the simulated US population would need to be fully vaccinated to achieve $R_{c}\;$< 1 given the low, baseline, and high vaccine effectiveness, respectively (Figure 4). The required fractions of fully vaccinated individuals to reduce the value of $R_{c}$ lower than one were 57%, 56%, and 54% under low levels of transmission and increased to near-saturated 82%, 79%, and 74% under high levels of transmission. We also obtained the $R_{c}$ for asymptomatic infections, which generally showed higher requirements of vaccinated individuals due to the lower vaccine effectiveness against asymptomatic infections (Figure S9). 

The assumed vaccine effectiveness ($\varepsilon_{L2}$) parameter under each scenario is denoted on top of each panel and is inferred based on real-world data of individuals receiving two doses of the BNT162b2 vaccine [27]. The heatmaps in the left row consider a low transmission rate, the heatmaps in the center row panels consider a baseline transmission rate, and the heatmaps in the right row panels consider a high transmission rate.

**Figure 5:**
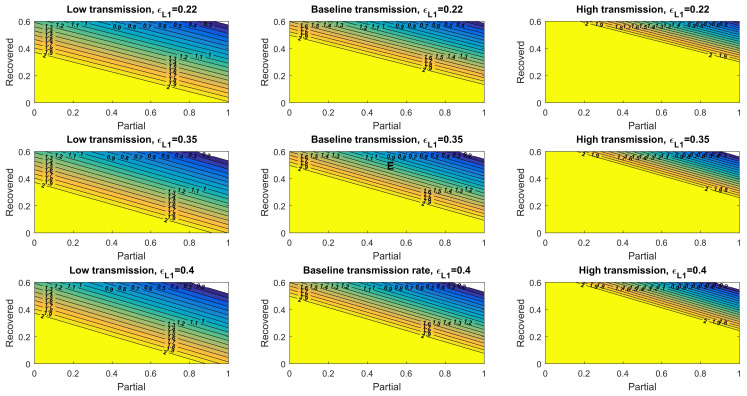
Control reproduction number for symptomatic transmission considering partial immunity and recovered individuals. The assumed vaccine effectiveness ($\varepsilon_{L1}$) parameter under each scenario is denoted on top of each panel and is inferred based on real-world data of individuals acquiring partial immunity as those induced by one dose of the BNT162b2 vaccine [27]. The heatmaps in the left row consider a low transmission rate, the heatmaps in the center row panels consider a baseline transmission rate, and the heatmaps in the right row panels consider a high transmission rate.

To model another scenario where the vaccine effectiveness reduces further due to factors such as a more resistant virus variant (e.g., omicron), we evaluated $R_{c}$ using the vaccine effectiveness parameters associated with one dose of the vaccine. The disease-free equilibrium in this population is composed of susceptible, recovered, and partially immune (equivalent to one dose vaccine’s immunity) individuals. Under a baseline transmission rate, and given that 30% of the population recovered from the disease and acquired natural immunity, impossible fractions of the simulated US population (over 100%) would need to have vaccine-induced, partial immunity to achieve $R_{c}$ < 1, given low, baseline, and high vaccine effectiveness, respectively (Figure 5). Even under low levels of transmission, the required fractions of vaccinated individuals reached >100%, 96%, and 92% under low levels of transmission (Figure 5). Thus, at this reduced level of vaccine effectiveness, a combination of measures to lower virus transmission would be required in conjunction with natural and vaccine-induced immunity to diminish the pandemic. Additionally, individuals will need higher levels of immunity (e.g., receive more vaccine doses) to reduce the control reproduction number below 1. 

### 3.5. Sensitivity Analyses of Model Parameters Affecting the Pandemic Trajectories

**Figure 6:**
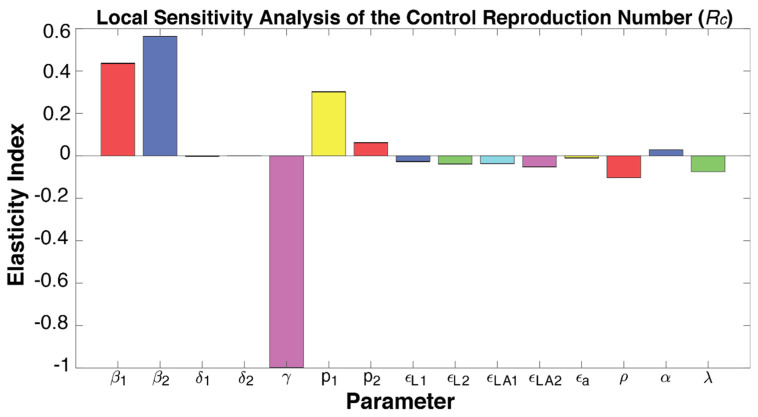
Elasticity index showing each modeled parameter’s effect on the control reproduction number, as determined by the local sensitivity analysis. For each column, a positive value represents an increase of the parameter is associated with an increment in the control reproduction number, whereas a negative value means a decrease.

To identify the transmission and vaccination factors that may affect the spread of the delta variant, we conducted sensitivity analyses [33] to determine how changes in each modeled parameter may alter the output $R_{c}$. In the local sensitivity analysis, the elastic index of each parameter was obtained by applying its partial derivatives and the substitution of its value one at a time (Material and Methods). As expected, increasing the force of infection for symptomatic ($\beta_{1}$) and asymptomatic ($\beta_{2}$) infections, along with the proportions of symptomatic individuals in unvaccinated and vaccine infections $\left(p_{1}\;\mathrm{and}\;p_{2}\right)$, are implicated in increasing the $R_{c}$ related to symptomatic infections. On the other hand, a higher recuperation rate (γ) could significantly reduce $R_{c}$, likely due to the natural immunity acquired by infected individuals. Increases in the vaccine effectiveness parameters for both one or two doses, as well as increasing the vaccination rate (ρ), would lower the $R_{c}$ and help control the pandemic (Figure 6). A higher relative transmission rate for individuals with only one dose $\mu_{1}$ is implicated by increasing the $R_{c}$ at a magnitude approximately twice of that for fully vaccinated individuals ($\mu_{2}$), implicating that the same increase in the transmission rate of partial-immunity individuals may spread more infections than that of fully vaccinated individuals.

**Figure 7:**
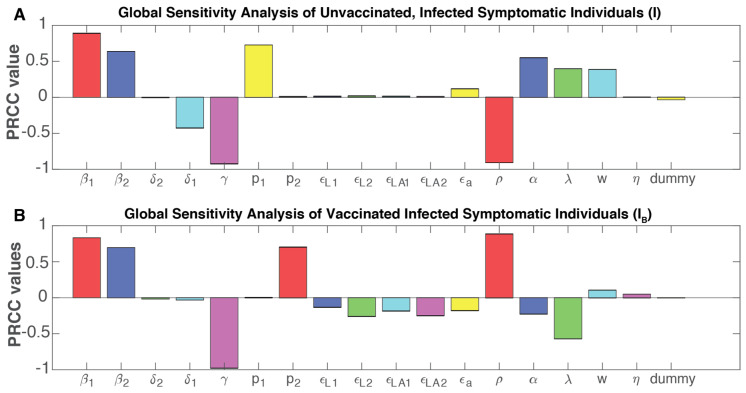
Global sensitivity analyses of the parameters correlated with the symptomatic infections in (**A**) unvaccinated and (**B**) vaccinated individuals. The relationship between each parameter and the infected subpopulation is measured by the partial rank correlation coefficient (PRCC); a positive PRCC indicates an increase in the parameters is correlated with an increase of the said sub-population, and a negative PRCC vice versa. The significance level of each PRCC is indicated in Figure S6.

While the local sensitivity analysis can help determine each factor’s impact one at a time, the input parameters can show drastic changes or spontaneously shape the model behavior and outputs based on their interactions. To model their concerted impact on the number of symptomatic infections in both vaccinated and unvaccinated individuals, we used a global sensitivity approach that computes the partial rank correlation coefficient (PRCC) by sampling with the Latin hypercube method [34]. For symptomatic infections in the unvaccinated individuals, the force of infection $\left(\beta_{1}\mathrm{and}\;\beta_{2}\right)$ is positively correlated with increasing the dynamics for this population (Figure 7A), and it is statically significant (Figure S10A). The recuperation rate (γ) is negatively correlated with newly infected cases, likely due to the natural immunity developed in recovered individuals. As the analyses focused on symptomatic individuals, $p_{1},$ by definition, shows a significant positive correlation (Figure 7A and Figure S9A). The model parameters that determine asymptomatic infections in unvaccinated individuals behave in a similar manner as symptomatic infections (Figure S11A,B). We also conducted global sensitivity analyses to identify how parameter changes may affect the subpopulations with partial or full vaccine-induced immunity. Partial immunity individuals are influenced negatively by the force of infection for symptomatic and asymptomatic infections, as well as $p_{1},$ indicating the fraction of symptomatic infections, while the recuperation rate is positively correlated with partial-immunity individuals (Figure S12A,B). Full-immunity individuals, derived from partial-immunity individuals who proceed to receive the second vaccination shot, are influenced in a similar manner (Figure S13A,B).

As increasing fractions of the US and global population become vaccinated, we further conducted global sensitivity analyses to identify the parameters associated with diminishing symptomatic infections in vaccinated individuals (i.e., breakthrough cases). Like symptomatic infections in unvaccinated individuals, breakthrough cases are positively correlated with the forces of infection for either symptomatic or asymptomatic individuals and negatively correlated with the recuperation rate (γ) (Figure 7B). As expected, each of the vaccine effectiveness parameters is negatively correlated with breakthrough cases. A higher vaccination rate is correlated with increased breakthrough cases, given it results in a larger pool of vaccinated individuals, but it also shows a strong negative correlation with infections in unvaccinated individuals, which can be ~10-fold higher (Figure 3). On the other hand, a high waning rate (α) for vaccine-induced immunity is correlated with a sizable increase in infections in unvaccinated individuals but reduced infections in vaccinated individuals, likely due to the model’s turnover of vaccinated individuals back to susceptible individuals upon complete immune waning. 

## 4. Discussion

Mathematical models can forecast the spread of infectious diseases and help inform public health decisions. Here, we developed a compartmentalized model that considers virus spreading in unvaccinated and vaccinated subpopulations to better portray the ongoing COVID-19 pandemic. Our application of the model to the US population considers the transmission and immunity parameters associated with the delta variant, a two-dose vaccination scheme, and the variant’s partial resistance to the vaccine that contributed to breakthrough cases, in addition to infections in unvaccinated individuals. 

Recently developed models from other groups have also distinguished the infection dynamics of the vaccinated and unvaccinated subpopulations in the COVID-19 pandemic of Mexico, Brazil, and Israel [23,35]. In comparison, we obtained parameters from real-world data to specifically stimulate the pandemic trajectory in the US and also included several parameters [24] that were not considered by these studies. For example, References [23,35] included the vaccine effectiveness evaluated in earlier clinical trials, whereas our model included vaccine effectiveness based on real-world data pertaining to the delta variant. We also included a parameter to account for the minor fraction of deaths that may still occur in vaccinated individuals [19] that were not considered by some of these studies [23,35]. 

Over 60% of the US population was fully vaccinated by the end of November 2021. Despite the larger fraction of the vaccinated compared to unvaccinated individuals, the model showed that the number of infected individuals would be a magnitude higher (~10×) in the unvaccinated compared to the vaccinated subpopulation within the ranges of likely transmission rates and vaccine effectiveness. Furthermore, deaths due to infection would also be significantly higher (~1.66×) in the unvaccinated compared to the vaccinated subpopulation. Vaccinating a larger fraction of the population, in conjunction with practicing NPIs, will continue to be one of the most effective means to diminish SARS-CoV-2 transmission [36]. Vaccine-induced immunity provided by two doses is more effective compared to one dose to reduce disease transmission or COVID-19-related hospitalizations/deaths [37]. Meanwhile, the reduction in vaccine effectiveness against virus variants and waning immunity may require additional solutions. We note that the vaccine waning parameter used herein is approximated, and while there is evidence supporting the loss of protection, the compounding effects of more infectious variants (i.e., delta and omicron) and waning immunity can be difficult to dissect [38]. Nevertheless, the rapid development of new vaccines and/or the administration of a third booster dose [39] are active areas of research that may yield promising results for controlling the pandemic.

The development of the model herein was informed by multiple parameters of virus transmission and vaccine-induced immunity, which resulted from consistent monitoring and the rapid sharing of data throughout the COVID-19 pandemic. For example, the model’s case counts and vaccination rates were established using the repository data of daily new COVID-19 infections and daily vaccination doses applied in the US [20]. The parameters of the BNT162b2 vaccine’s immunity against the delta variant (either one or two doses) were obtained from the real-world data [27] determined in the UK, which, when applied to the US pandemic, allowed us to circumvent the circular logic of forecasting the pandemic using vaccine effectiveness parameters derived from the same population. Scientific progress in the ongoing COVID-19 pandemic is accelerated by the promptness and transparency in data sharing practices, which could also help tackle a wide array of public health challenges.

Our model has several limitations, some of which are addressed in other mathematical models developed during this pandemic. First, our model does not consider the population heterogeneity in the transmission dynamics, which has been shown to reduce the required number of vaccinated individuals to achieve herd immunity [40]. Second, we do not consider seasonality [41], although the seasonal trend for SARS-CoV-2 remains unclear at this point. Third, nonreported SARS-CoV-2 infections were not included in the model and may vary based on the ascertainment rates. Fourth, projecting the complex COVID-19 pandemic and accounting for multiple dynamic parameters with uncertainties, (e.g., how many people would become unvaccinated and newly arising variants like omicron) is challenging. Inaccurate parameters, including those portraying vaccine and transmission dynamics, will inevitably lead to inaccurate models projections, especially when the model is highly complex. Our model may not provide the exact projections as the parameter fluctuates in real-world scenarios, but the modeled behaviors may suggest trends under different vaccination effectiveness and transmission rates as a reference. Finally, while the analyses herein utilized the immunity parameters for one/two doses of the BNT162b2 vaccine and the delta variant, our model can be applied to model other vaccines/doses and emerging virus strains (i.e., omicron) in different populations.

Countries (e.g., Taiwan [42] and New Zealand [43]) that have successfully contained the COVID-19 pandemic have demonstrated the importance of using nonpharmaceutical interventions (NPIs) and contact tracing in addition to vaccination. Other mathematical models have demonstrated that combining NPI and vaccination roll-outs will help reduce the control reproduction number (Rc) in the UK, Italy, and Portugal [44,45,46]. The model developed by Moore et al. 2021 [43] demonstrated the need for NPI in the context of the alpha variant, which has been largely replaced by the delta variant in 2021. Giordano et al. 2021 [44] modeled NPI with an open and close strategy to avoid economic losses, finding that NPIs may have a greater capacity to reduce deaths and improve economic outcomes than vaccination campaigns in the Italian pandemic. Aligned with these results, Viana et al. 2021 [45] demonstrated the need for NPI to reduce the spread of the virus. However, none of the aforementioned models included a SARS-CoV-2 variant as transmissible as delta. Most of the NPI used in these models were of restrictions of social interactions, yet prolonged social distancing may also negatively impact the economy and mental health [47,48]. Meanwhile, the use of face masks can effectively reduce the spread of the virus [49]; for example, a mathematical model showed that wearing face masks can reduce transmission in a partially vaccinated school setting [50]. 

While vaccines successfully reduced hospitalizations and deaths in the initial “honeymoon period” [51], COVID-19 cases have increased again in multiple countries due to the newly emerged delta and omicron variants. While transmission also occurs in vaccinated individuals, the immunity provided by a vaccine can reduce viral loads [52]. Higher transmission rates and viral loads provide more opportunities for mutagenesis [53], and VOCs capable of higher transmission rates and immune evasion may continue to arise [54,55,56]. At the time of writing, most VOCs have not shown significantly higher transmission compared to the delta variant or its sublineages [57], but this may change if the transmission rate remains high (i.e., the omicron variant). Tempering the spread of SARS-CoV-2 variants will require enhanced global efforts on sequencing and variant detection, establishing reproducible analysis pipelines and the rapid sharing of data across geographical boundaries [58]. Given that asymptomatic individuals can spread SARS-CoV-2 [56,59,60], increased testing and surveillance will also be critical. 

## 5. Conclusions

In summary, our mathematical model provides a projection of the ongoing COVID-19 pandemic in the US, distinguishing new cases arising from unvaccinated vs. in vaccinated individuals. While the results may not provide the exact forecast, the behavior of the model can inform public health decisions. For example, the significantly higher magnitude of new cases arising from unvaccinated population highlights the need to vaccinate as large a fraction of the population as possible, and the effect of waning immunity in the sensitivity analysis suggests the importance of booster vaccine doses. Overall, an enhanced vaccination roll-out in conjunction with the extensive contact tracing and practice of NPIs, such as wearing face masks, will be instrumental to reducing the spread and further mutagenesis of the SARS-CoV-2 virus.

## Data Availability

The original contributions presented in the study are included in the article/Supplementary Materials. All code used for the analysis is available at https://github.com/UgoAvila/Delta-Variant-In-the-US (last accessed on 23 December 2021).

## Supplementary Materials

The following are available online at www.mdpi.com/article/10.3390/v14010158/s1: Figure S1: Fitting and projecting the vaccination rate, Figure S2: Projection of the cumulative counts of recuperated and deceased individuals based on different transmission and vaccination rates, Figure S3: Modeled projections of asymptomatic infections in unvaccinated and vaccinated subpopulations under different vaccinate rates and vaccine effectiveness, Figure S4: Modeled projections of baseline vaccinated rate of symptomatic infections in unvaccinated and vaccinated subpopulations under different transmission rates and vaccine effectiveness, Figure S5: Modeled projections of high vaccinated rate of symptomatic infections in unvaccinated and vaccinated subpopulations under different transmission rates and vaccine effectiveness, Figure S6: Modeled projections of asymptomatic infections in unvaccinated and vaccinated subpopulations under different vaccination rates and vaccine effectiveness, Figure S7: Modeled projections of cumulative death in the US population under different vaccination rates and vaccine effectiveness, Figure S8: Reproduction Control Number of the delta variant considering full immunity and recovered individuals for asymptomatic infections, Figure S9: Significance levels of the partial rank correlation coefficient (PRCC, depicted in Figure 6) for each parameter from the global sensitivity analyses, Figure S10: Global sensitivity analysis of the parameters correlated with the unvaccinated, infected asymptomatic individuals in the mathematical model, Figure S11: Global sensitivity analysis of the parameters correlated with the partially vaccinated individuals (one dose) in the mathematical model, Figure S12: Global sensitivity analysis of the parameters correlated with the fully vaccinated individuals (two doses) in the mathematical model, Figure S13: Global sensitivity analyses of the parameters correlated with the fully vaccinated individuals (two doses) in the mathematical model, Table S1: Parameters used in the simulation of our mathematical model, and Table S2: US vaccination rates in the relevant time ranges.

## Appendix A

### Appendix A.1. Mathematical Model with Asymptomatic Individuals and Vaccination

We develop and apply a compartmental differential equation to understand the dynamics of the spread of the delta variant in vaccinated and non-vaccinated individuals. The mathematical model contains two separate sets of equations for the virus strains that affect the same susceptible population and vaccinated subpopulation. Our SPFEIARD model evaluates the dynamics of ten subpopulations at any given time t, which are denoted as: $S\left(t\right),\;E_{1}\left(t\right)$, etc. Figure 1 shows a diagram of the flow through the compartmentalized subpopulations.

Susceptible population *S*(*t*): We do not consider any natural recruitments (births), *t* in this model. The susceptible population decreases when they interact with a symptomatic delta variant infection at a rate $\beta_{1}$ and an asymptomatic delta variant infection at a rate $\beta_{2}$. The susceptible population may increase at a rate α associated with the waning rate of a vaccine, a parameter of an imperfect vaccine. Finally, the susceptible population will be vaccinated at a rate ρ≥0, multiplied by the all or nothing protection of a vaccine $\left(1-\varepsilon_{a}\right)$. The rate of change of the susceptible population is expressed in the following equation:

(A1) $$\frac{dS}{dt}=-\beta_{1}\left(\frac{I+I_{B}}{N}\right)S-\beta_{2}\left(\frac{A+A_{B}}{N}\right)S+\alpha V+\eta R-\left(1-\varepsilon_{a}\right)\rho S.$$

Unvaccinated population exposed to the delta variant, E(t): Since we are simulating an infection caused by a virus, we need to include the exposed population. This subpopulation are individuals that are infected but not infectious yet. It increases at a rate of infection $\beta_{1}$ and $\beta_{2}$ for symptomatic and asymptomatic infection from the delta variant. This subpopulation evaluated the dynamics of new infections of individuals that have not receive any type of vaccine. This population decreases at a rate w, which denotes the average length of the latent period. Hence,

(A2) $$\frac{dE}{dt}=\left(\frac{\beta_{1}I}{N}\right)S+\left(\frac{\beta_{2}A}{N}\right)S-wE.$$

Unvaccinated population infected by the delta variant, I(t): Infected individuals that develop symptoms for the delta variant are generated at a proportion $p_{1}\in \left(0,1\right)$ from the exposed non-vaccinated subpopulation. They recover at a rate γ and die from the disease at a rate $\delta_{1}$. Consequently,

(A3) $$\frac{dI}{dt}=p_{1}wE-\left(\delta_{1}+\gamma \right)I.$$

Asymptomatic, unvaccinated population infected by the delta variant, A(t): This population is considered an infected population but do not develop symptoms of COVID-19. They are important to include in our model, because they can spread the virus, and they are generated at a proportion $\left(1-p_{1}\right)$ from the exposed class. This population recovers at a rate γ. This population does not die from this disease, because they do develop symptoms. Therefore,

(A4) $$\frac{dA}{dt}=\left(1-p_{1}\right)wE-\gamma A.$$

Population with partial immunity (one dose), P(t): This subpopulation is vaccinated at a rate ρ≤0 multiplied by the all or nothing protection $\varepsilon_{a}$. This population decreases at a rate $\left(1-\varepsilon_{L1}\right)\beta_{1}$ due to the imperfect or vaccine efficiency provided by one dose of the Pfizer vaccine for symptomatic individuals. The vaccine efficiency protections extend to the asymptomatic infected individuals as well, so it decays at a rate $\left(1-\varepsilon_{LA1}\right)\beta_{2}$. Finally, this population decreases when individuals receive the second dose of the Pfizer vaccine at a rate λ. Hence,

(A5) $$\frac{dP}{dt}=\left(1-\varepsilon_{a}\right)\rho S-\left(\frac{\left(1-\varepsilon_{L1}\right)\beta_{1}\left(I+(\mu_{1}*I_{B})\right)}{N}\right)P-\left(\frac{\left(1-\varepsilon_{LA1}\right)\beta_{2}\left(A+(\mu_{1}*A_{B})\right)}{N}\right)P-\lambda P.$$

Population with full immunity (two doses), V(t): This population increases at a rate λ, but for it to increase, it is mandatory that individuals are first part of the partial immunity subpopulation. Due to the imperfect vaccine, this population decays at a rate $\left(1-\varepsilon_{L2}\right)\beta_{1}$ for symptomatic infected individuals due to the interaction with individuals infected with the delta variant. Additionally, it decays at a rate $\left(1-\varepsilon_{LA2}\right)\beta_{2}$ for asymptomatic infected individuals due to the interaction with individuals infected with the delta variant. Finally, the population decays as well at a rate α due to the loss of protection or waning immunity provided by the protection of the vaccine.

(A6) $$\frac{dV}{dt}=\lambda P-\left(\frac{\left(1-\varepsilon_{L2}\right)\beta_{1}\left(I+(\mu_{2}*I_{B})\right)}{N}\right)V-\left(\frac{\left(1-\varepsilon_{LA2}\right)\beta_{2}\left(A+(\mu_{2}*A_{B})\right)}{N}\right)V-\alpha V.$$

Vaccinated population exposed to the delta variant (one or two doses), s, $E_{B}\left(t\right)$: We include the exposed vaccinated population to the delta variant, because we are modeling a virus. The population enlarges at a rate $\beta_{1}$ for symptomatic infected individuals and at a rate $\beta_{2}$ for asymptomatic individuals, both from the delta variant. Moreover, vaccinated individuals who interact with symptomatic or asymptomatic unvaccinated indivduals are incorporated at rates $\left(1-\varepsilon_{L1}\right)$ and $\left(1-\varepsilon_{LA1}\right),$ respectively, considering the vaccine efficiencies of the vaccine to the delta variant for one dose. Additionally, vaccinated individuals who interact with symptomatic or asymptomatic vaccinated indivduals are incorporated at rates $\left(1-\varepsilon_{L2}\right)$ and $\left(1-\varepsilon_{LA2}\right),$ respectively. This subpopulation deceases at a rate w, the length where individuals pass from being infected to being both infected and infectious. Hence,

(A7) $$\frac{dE_{B}}{dt}=\left(\frac{\left(1-\varepsilon_{L1}\right)\beta_{1}\left(I+(\mu_{1}*I_{B})\right)}{N}\right)P+\left(\frac{\left(1-\varepsilon_{LA1}\right)\beta_{2}\left(A+(\mu_{1}*A_{B})\right)}{N}\right)P+\left(\frac{\left(1-\varepsilon_{L2}\right)\beta_{1}\left(I+(\mu_{2}*I_{B})\right)}{N}\right)V\;+\left(\frac{\left(1-\varepsilon_{LA2}\right)\beta_{2}\left(A+(\mu_{2}*A_{B})\right)}{N}\right)V-wE_{B}.$$

Vaccinated population infected by the delta variant, $I_{B}\left(t\right)$: For all individuals that are infected while being vaccinated regardless of the dose received, we assume they develop symptoms at a proportion $p_{2}\in \left(0,1\right)$, recover at a rate γ, and die at a rate $\delta_{2}$. Consequently,

(A8) $$\frac{dI_{B}}{dt}=p_{2}wE_{B}-\left(\delta_{2}+\gamma \right)I_{B}.$$

Asymptomatic, vaccinated population infected by the delta variant, $A_{B}\left(t\right)$: Individuals that are infected while being vaccinated regardless of the dose received but do not develop symptoms at a proportion $\left(1-p_{2}\right)$ from the exposed subpopulation. They recover at a rate γ. Therefore,

(A9) $$\frac{dA_{B}}{dt}=\left(1-p_{2}\right)wE_{B}-\gamma A_{B}.$$

Recovered population, R(t): All individuals infected (vaccinated or not, with symptoms or not) will recover at a rate γ from the delta variant. We acknowledge in this population the loss of protection from natural immunity at a rate η. Consequently,

(A10) $$\frac{dR}{dt}=\gamma \left(I+A+I_{B}+A_{B}\right)-\eta R.$$

Deceased population, D(t): Infected, unvaccinated individuals with symptoms decease at a rate $\delta_{1}$ from the delta variant. Infected, vaccinated individuals decease at a rate $\delta_{2}$. Therefore,

(A11) $$\frac{dD}{dt}=\delta_{1}I_{1}+\delta_{2}I_{B}\;.$$

Henceforth, the system of differential equations that will simulate the dynamics of the original strain and the delta variant with vaccination is:

(A12) $$\frac{dS}{dt}=-\beta_{1}\left(\frac{I+I_{B}}{N}\right)S-\beta_{2}\left(\frac{A+A_{B}}{N}\right)S+\alpha F+\eta R-\left(1-\varepsilon_{a}\right)\rho S,\;\frac{dE}{dt}=\left(\frac{\beta_{1}I}{N}\right)S+\left(\frac{\beta_{1}A}{N}\right)S-wE,\;\frac{dI}{dt}=p_{1}wE-\left(\delta_{1}+\gamma \right)I,\;\frac{dA}{dt}=\left(1-p_{1}\right)wE-\gamma A,$$

$$\frac{dP}{dt}=\left(1-\varepsilon_{a}\right)\rho S-\left(\frac{\left(1-\varepsilon_{L1}\right)\beta_{1}\left(I+(\mu_{1}*I_{B})\right)}{N}\right)P-\left(\frac{\left(1-\varepsilon_{LA1}\right)\beta_{2}\left(A+(\mu_{1}*A_{B})\right)}{N}\right)P-\lambda P,\;\frac{dV}{dt}=\lambda P-\left(\frac{\left(1-\varepsilon_{L2}\right)\beta_{1}\left(I+(\mu_{2}*I_{B})\right)}{N}\right)V-\left(\frac{\left(1-\varepsilon_{LA2}\right)\beta_{2}\left(A+(\mu_{2}*A_{B})\right)}{N}\right)V-\alpha V,\;\begin{array}{cc} \frac{dE_{B}}{dt}= & \left(\frac{\left(1-\varepsilon_{L1}\right)\beta_{1}\left(I+(\mu_{1}*I_{B})\right)}{N}\right)P+\left(\frac{\left(1-\varepsilon_{LA1}\right)\beta_{2}\left(A+(\mu_{1}*A_{B})\right)}{N}\right)P\\ & +\left(\frac{\left(1-\varepsilon_{L2}\right)\beta_{1}\left(I+(\mu_{2}*I_{B})\right)}{N}\right)V+\left(\frac{\left(1-\varepsilon_{LA2}\right)\beta_{2}\left(A+(\mu_{2}*A_{B})\right)}{N}\right)V-wE_{B}, \end{array}\;\frac{dI_{B}}{dt}=p_{2}wE_{B}-\left(\delta_{2}+\gamma \right)I_{B},\;\frac{dA_{B}}{dt}=\left(1-p_{2}\right)wE_{B}-\gamma A_{B},\;\frac{dR}{dt}=\gamma \left(I+A+I_{B}+A_{B}\right)-\eta R,\;\frac{dD}{dt}=\delta_{1}I_{1}+\delta_{2}I_{B}.$$

We can derive that N(t) at a time t is given by,

$$N\left(t\right)=S\left(t\right)+E\left(t\right)+I\left(t\right)+A\left(t\right)+P\left(t\right)+V\left(t\right)+E_{B}\left(t\right)+I_{B}\left(t\right)+A_{B}\left(t\right)+R\left(t\right)+D\left(t\right)$$

### Appendix A.2. Control Reproduction Number with a Disease-Free Equilibrium

The entire derivation of $R_{c}$ is as follows:

$$ℱ=\left[\begin{array}{c} \left(\frac{\beta_{1}I}{N}\right)S+\left(\frac{\beta_{1}A}{N}\right)S\\ 0\\ \begin{array}{c} 0\\ \left(\frac{\left(1-\varepsilon_{L1}\right)\beta_{1}\left(I+(\mu_{1}*I_{B})\right)}{N}\right)P+\left(\frac{\left(1-\varepsilon_{LA1}\right)\beta_{2}\left(A+(\mu_{1}*A_{B})\right)}{N}\right)P+\left(\frac{\left(1-\varepsilon_{L2}\right)\beta_{1}\left(I+(\mu_{2}*I_{B})\right)}{N}\right)V+\left(\frac{\left(1-\varepsilon_{LA2}\right)\beta_{2}\left(A+(\mu_{2}*A_{B})\right)}{N}\right)V\\ \begin{array}{c} 0\\ 0 \end{array} \end{array} \end{array}\right].$$

The derivative of ℱ at $x_{DF}$ is:

$$F=\left[\begin{array}{ccc} 0 & \frac{\beta_{1}S^{*}}{N^{*}} & \frac{\beta_{1}S^{*}}{N^{*}}\\ 0 & 0 & 0\\ \begin{array}{c} 0\\ 0\\ \begin{array}{c} 0\\ 0 \end{array} \end{array} & \begin{array}{c} 0\\ \frac{\left(1-\varepsilon_{L1}\right)\mu_{1}\beta_{1}P^{*}}{N^{*}}+\frac{\left(1-\varepsilon_{L2}\right)\mu_{2}\beta_{1}V^{*}}{N^{*}}\\ \begin{array}{c} 0\\ 0 \end{array} \end{array} & \begin{array}{c} 0\\ \frac{\left(1-\varepsilon_{LA1}\right)\beta_{2\mu_{1}}P^{*}}{N^{*}}+\frac{\left(1-\varepsilon_{LA2}\right)\mu_{2}\beta_{2}V^{*}}{N^{*}}\\ \begin{array}{c} 0\\ 0 \end{array} \end{array} \end{array}\begin{array}{ccc} 0 & \frac{\beta_{1}S^{*}}{N^{*}} & \frac{\beta_{2}S^{*}}{N^{*}}\\ 0 & 0 & 0\\ \begin{array}{c} 0\\ 0\\ \begin{array}{c} 0\\ 0 \end{array} \end{array} & \begin{array}{c} 0\\ \frac{\left(1-\varepsilon_{L1}\right)\beta_{1}\mu_{1}P^{*}}{N^{*}}+\frac{\left(1-\varepsilon_{L2}\right)\beta_{1}\mu_{2}V^{*}}{N^{*}}\\ \begin{array}{c} 0\\ 0 \end{array} \end{array} & \begin{array}{c} 0\\ \frac{\left(1-\varepsilon_{LA1}\right)\beta \mu_{1}{}_{2}P^{*}}{N^{*}}+\frac{\left(1-\varepsilon_{LA2}\right)\mu_{1}\beta_{2}V^{*}}{N^{*}}\\ \begin{array}{c} 0\\ 0 \end{array} \end{array} \end{array}\right].$$

The transition matrix V is:

$$V=\left[\begin{array}{c} wE\\ -p_{1}wE+\left(\gamma +\delta_{1}\right)I\\ \begin{array}{c} -\left(1-p_{1}\right)wE+\gamma A\\ wE_{B}\\ \begin{array}{c} -p_{2}wE_{B}+\left(\gamma +\delta_{2}\right)I_{B}\\ -\left(1-p_{2}\right)wE_{B}+\gamma A_{B} \end{array} \end{array} \end{array}\right].$$

The derivative of V at $x_{DF}$ is:

$$V=\left[\begin{array}{c} w\\ -p_{1}w\\ \begin{array}{c} -(1-p_{1})w\\ 0\\ \begin{array}{c} 0\\ 0 \end{array} \end{array} \end{array}\begin{array}{c} 0\\ \gamma +\delta_{1}\\ \begin{array}{c} 0\\ 0\\ \begin{array}{c} 0\\ 0 \end{array} \end{array} \end{array}\begin{array}{c} 0\\ 0\\ \begin{array}{c} \gamma \\ 0\\ \begin{array}{c} 0\\ 0 \end{array} \end{array} \end{array}\begin{array}{c} 0\\ 0\\ \begin{array}{c} 0\\ w\\ \begin{array}{c} -p_{2}w\\ -\left(1-p_{2}\right)w \end{array} \end{array} \end{array}\begin{array}{c} 0\\ 0\\ \begin{array}{c} 0\\ 0\\ \begin{array}{c} \gamma +\delta_{2}\\ 0 \end{array} \end{array} \end{array}\begin{array}{c} 0\\ 0\\ \begin{array}{c} 0\\ 0\\ \begin{array}{c} 0\\ \gamma \end{array} \end{array} \end{array}\right].$$

The inverse of *V* is:

$$V^{-1}=\left[\begin{array}{cccccc} \frac{1}{w} & 0 & 0 & 0 & 0 & 0\\ -\frac{p_{1}}{\left(\gamma +\delta_{1}\right)} & \frac{1}{\left(\gamma +\delta_{1}\right)} & 0 & 0 & 0 & 0\\ -\frac{\left(1-p_{1}\right)}{\gamma } & 0 & \frac{1}{\gamma } & 0 & 0 & 0\\ 0 & 0 & 0 & \frac{1}{w} & 0 & 0\\ 0 & 0 & 0 & -\frac{p_{2}}{\left(\gamma +\delta_{2}\right)} & \frac{1}{i} & 0\\ 0 & 0 & 0 & -\frac{\left(1-p_{2}\right)}{\left(\gamma +\delta_{2}\right)} & 0 & \frac{1}{\gamma } \end{array}\right].$$

It follows that

$$FV^{-1}=\left[\begin{array}{cccccc} K & \frac{\beta_{1}S^{*}}{N^{*}\left(\gamma +\delta_{1}\right)} & \frac{\beta_{2}S^{*}}{N^{*}\gamma } & A & \frac{\beta_{1}S^{*}}{N^{*}\left(\gamma +\delta_{1}\right)} & \frac{\beta_{2}S^{*}}{N^{*}\gamma }\\ 0 & 0 & 0 & 0 & 0 & 0\\ 0 & 0 & 0 & 0 & 0 & 0\\ R & \frac{\left(1-\varepsilon_{L1}\right)\beta_{1}\mu_{1}P^{*}+\left(1-\varepsilon_{L2}\right)\beta_{1}\mu_{2}V^{*}}{N^{*}\left(\gamma +\delta_{1}\right)} & \frac{\left(1-\varepsilon_{LA1}\right)\beta_{2}\mu_{1}P^{*}+\left(1-\varepsilon_{LA2}\right)\mu_{2}\beta_{2}V^{*}}{N^{*}\gamma } & E & \frac{\left(1-\varepsilon_{L1}\right)\beta_{1}\mu_{1}P^{*}+\left(1-\varepsilon_{L2}\right)\mu_{1}\beta_{1}V^{*}}{N^{*}\left(\gamma +\delta_{2}\right)} & \frac{\left(1-\varepsilon_{LA1}\right)\mu_{1}\beta_{2}P^{*}+\left(1-\varepsilon_{LA2}\right)\beta_{2}\mu_{2}V^{*}}{N^{*}\gamma }\\ 0 & 0 & 0 & 0 & 0 & 0\\ 0 & 0 & 0 & 0 & 0 & 0 \end{array}\right].$$

where:

$$K=\frac{p_{1}\beta_{1}S^{*}}{N^{*}\left(\gamma +\delta_{1}\right)}+\frac{(1-p_{1})\beta_{2}S^{*}}{N^{*}\gamma }=\frac{S^{*}}{N^{*}}\left[\frac{p_{1}\beta_{1}}{\left(\gamma +\delta_{1}\right)}+\frac{(1-p_{1})\beta_{2}}{\gamma }\right],\;A=\frac{p_{2}\beta_{1}S^{*}}{N^{*}\left(\gamma +\delta_{2}\right)}+\frac{(1-p_{2})\beta_{2}S^{*}}{N^{*}\gamma }=\frac{S^{*}}{N^{*}}\left[\frac{p_{2}\beta_{1}}{\left(\gamma +\delta_{2}\right)}+\frac{(1-p_{2})\beta_{2}}{\gamma }\right],\;\begin{array}{cc} R= & \frac{\left(1-\varepsilon_{L1}\right)p_{1}\mu_{1}\beta_{1}P^{*}+\left(1-\varepsilon_{L1}\right)p_{1}\mu_{2}\beta_{1}V^{*}}{N^{*}\left(\gamma +\delta_{1}\right)}\\ & +\frac{(1-p_{1})\left(1-\varepsilon_{LA1}\right)\mu_{1}\beta_{2}P^{*}+(1-p_{1})\left(1-\varepsilon_{LA2}\right)\mu_{1}\beta_{2}V^{*}}{N^{*}\gamma }\\ & =\frac{\beta_{1}p_{1}}{\left(\gamma +\delta_{1}\right)}\left[\frac{(1-\varepsilon_{L1})\mu_{1}P^{*}}{N^{*}}+\frac{(1-\varepsilon_{L2})\mu_{2}V^{*}}{N^{*}}\right]\\ & +\frac{\beta_{2}\left(1-p_{1}\right)}{\gamma }\left[\frac{(1-\varepsilon_{LA1})\mu_{1}P^{*}}{N^{*}}+\frac{(1-\varepsilon_{LA2})\mu_{1}V^{*}}{N^{*}}\right], \end{array}\;\begin{array}{cc} E= & \frac{\left(1-\varepsilon_{L1}\right)\mu_{1}p_{2}\beta_{1}P^{*}+\left(1-\varepsilon_{L1}\right)\mu_{2}p_{2}\beta_{1}V}{N^{*}\left(\gamma +\delta_{2}\right)}\\ & +\frac{(1-p_{2})\left(1-\varepsilon_{LA1}\right)\mu_{1}\beta_{2}P^{*}+(1-p_{2})\left(1-\varepsilon_{LA2}\right)\mu_{2}\beta_{2}V^{*}}{N^{*}\gamma }\\ & =\frac{\beta_{1}p_{2}}{\left(\gamma +\delta_{2}\right)}\left[\frac{(1-\varepsilon_{L1})\mu_{1}P^{*}}{N^{*}}+\frac{(1-\varepsilon_{L2})\mu_{2}V^{*}}{N^{*}}\right]\\ & +\frac{\beta_{2}\left(1-p_{2}\right)}{\gamma }\left[\frac{(1-\varepsilon_{LA1})\mu_{1}P^{*}}{N^{*}}+\frac{(1-\varepsilon_{LA2})\mu_{2}V^{*}}{N^{*}}\right]. \end{array}$$

Hence the control reproduction number $R_{c}$ of model (A12) is:

(A13) $$R_{c}=\frac{S^{*}}{N^{*}}\left[\frac{p_{1}\beta_{1}}{\left(\gamma +\delta_{1}\right)}+\frac{(1-p_{1})\beta_{2}}{\gamma }\right]+\frac{\beta_{1}p_{2}}{\left(\gamma +\delta_{2}\right)}\left[\frac{(1-\varepsilon_{L1})\mu_{1}P^{*}}{N^{*}}+\frac{(1-\varepsilon_{L2})\mu_{2}V^{*}}{N^{*}}\right]\;+\frac{\beta_{2}\left(1-p_{2}\right)}{\gamma }\left[\frac{(1-\varepsilon_{LA1})\mu_{1}P^{*}}{N^{*}}+\frac{(1-\varepsilon_{LA2})\mu_{2}V^{*}}{N^{*}}\right]$$

### Appendix A.3. Control Reproduction Number for the Delta Variant

Equation (A12) represent the reproduction number for the delta variant in terms of the susceptible population, one-dose vaccinated individuals, and two-dose vaccinated individuals. One possible disease-free equilibrium is where we have suspectable individuals, a proportion of individuals who have received one dose (partial immunity), and a proportion of individuals with two-dose vaccination (full immunity). Consequently, a scenario of the disease-free equilibrium mentioned above is $x_{DF}=\left(S^{*},0,0,0,0,V^{*},0,0,0,R^{*},0\right),$ and the total population is

(A14) $$N^{*}=S^{*}+R^{*}+V^{*}.$$

Since we want to define a proportion of susceptible, vaccinated one-dose, and vaccinated two-dose individuals, we can divide Equation (14) by the total population. Therefore,

$$\frac{S^{*}}{N^{*}}+\frac{R^{*}}{N^{*}}+\frac{V^{*}}{N^{*}}=1,\;\frac{PR^{*}}{N^{*}}=x\;and\;\frac{V^{*}}{N^{*}}=y.$$

It follows that,

$$\frac{S^{*}}{N^{*}}=1-x-y.$$

We rewrite Equation (A13) with the expression mentioned above. Hence,

(A15) $$R_{c}=\left(1-x-y\right)\left[\frac{p_{1}\beta_{1}}{\left(\gamma +\delta_{1}\right)}+\frac{(1-p_{1})\beta_{2}}{\gamma }\right]+\frac{\beta_{1}p_{2}}{\left(\gamma +\delta_{2}\right)}\left[+(1-\varepsilon_{L2})\mu_{2}y\right]\;+\frac{\beta_{2}\left(1-p_{2}\right)}{\gamma }\left[+(1-\varepsilon_{LA2})\mu_{2}y\right]$$
