# Supplementary material for: Modeling the Transmission of the SARS-CoV-2 Delta Variant in a Partially Vaccinated Population
Source: Viruses. 2022 Jan 16;14(1):158. doi: 10.3390/v14010158 (PMC8781299; doi:10.3390/v14010158)
Supplement: Supplementary file 1 [file viruses-14-00158-s001.zip › viruses-1441122-supplementary.pdf]

Supplementary Materials for

Modeling the transmission of the SARS-CoV-2 delta variant in a partially vaccinated population

Ugo Avila-Ponce de León, Eric Avila-Vales, Kuan-lin Huang
Correspondence to: avila@correo.uady.mx; kuan-lin.huang@mssm.edu

This PDF file includes:

Figures. S1 to S13
Table S1 to S2

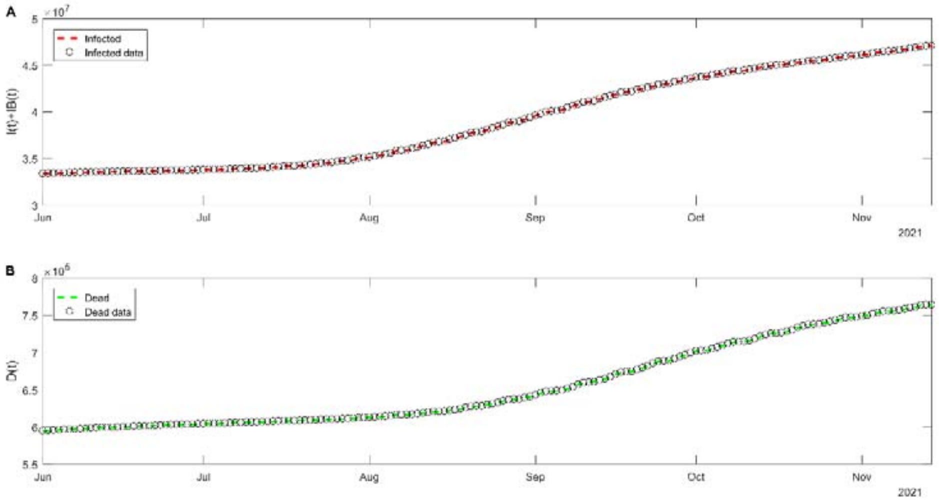

Figure. S1. Fitting the transmission and death rate. (A) Projection of the transmission rate of our model with the data obtained from the University of John Hopkins repository of COVID-19 data. (B) Projection of the death rate of our model with the data obtained from the University of John Hopkins repository of COVID-19 data.

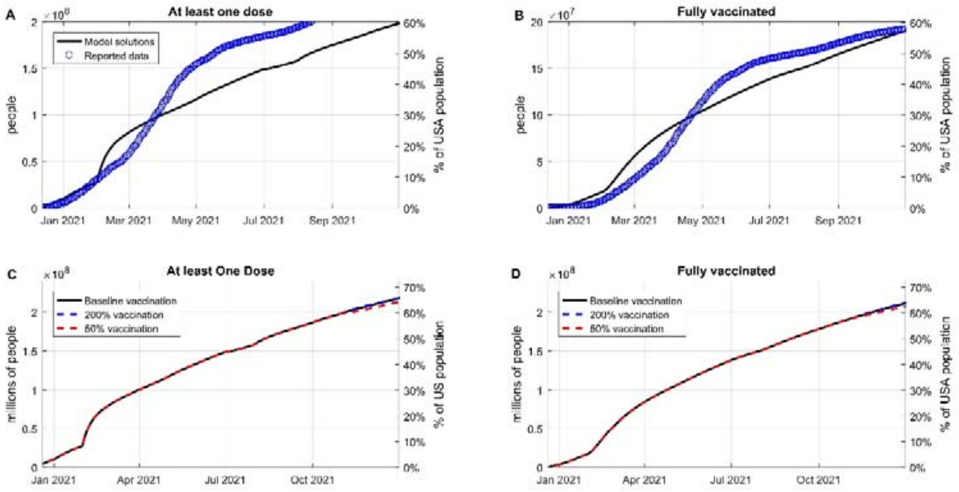

Figure. S2. Fitting and projecting the vaccination rate. (A) Deriving a function that describes the behavior of the daily doses applied in the US for one dose-vaccinated individuals. (B) Deriving a function that describes the behavior of the second dose application for individuals with one dose of vaccination. (C) Dynamics of individuals with at least one dose of vaccination considering the baseline vaccination rate since the modelled date, as well as situations where the vaccination rate is doubled or diminished 50%. (D) Dynamics of individuals with at two doses of vaccination considering the baseline vaccination rate since the modelled date, as well as situations where the vaccination rate is doubled or diminished 50%.

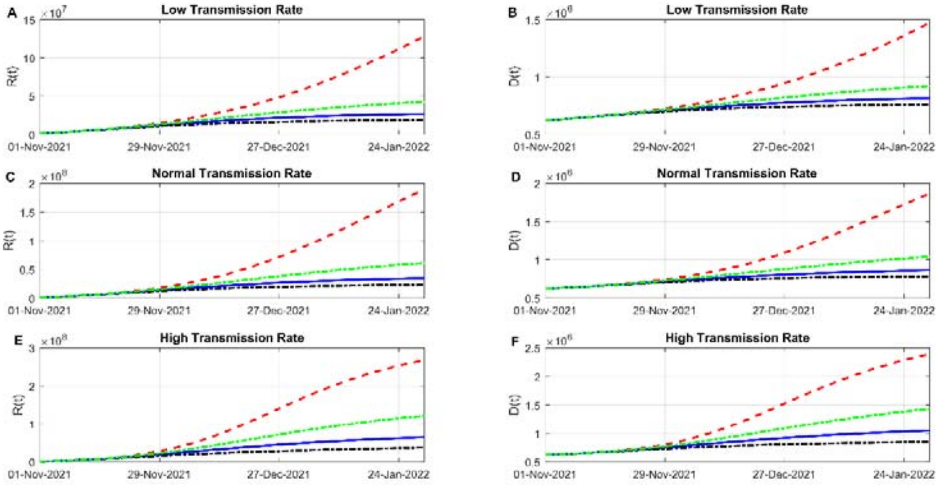

Figure. S3. Projection of the cumulative counts of recuperated and deceased individuals based on different transmission rates and vaccination rates, including (A) recuperated individuals and (B) deceased individuals under a low transmission rate; (C) recuperated individuals and (D) deceased individuals under a baseline transmission rate; (E) recuperated individuals and (F) deceased individuals under a high transmission rate. The red line presents zero vaccination; the green line represents a 50% decrease in VR; the blue dotted line means baseline VR and the black dotted line denotes 200% VR.

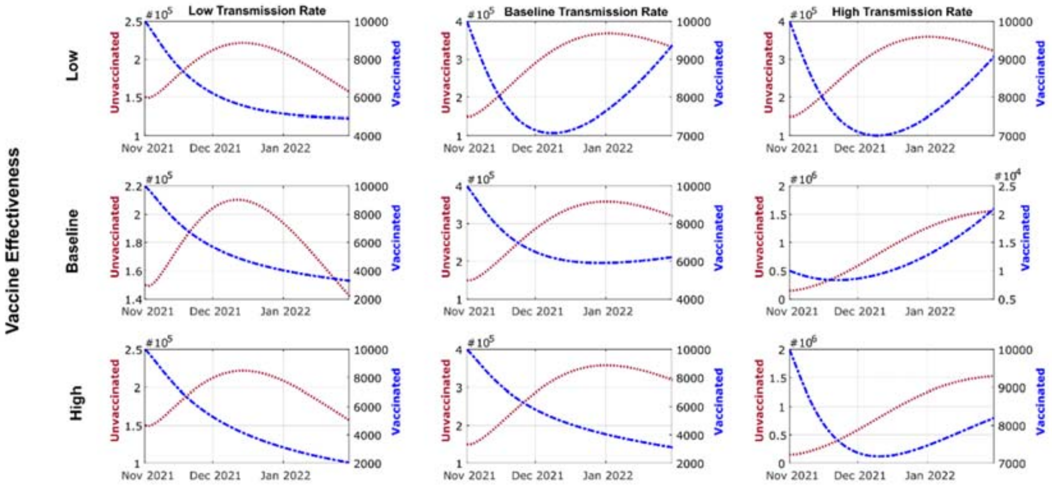

Figure. S4. Modelled projections of low vaccinated rate of symptomatic infections in unvaccinated and vaccinated subpopulations under different transmission rates and vaccine effectiveness. The low and high vaccination effectiveness denotes the lower bound and the higher bound of the 95% confidence intervals of the real-world data, respectively. Panels (A), (D), (G) represents scenarios with a low transmission rate, (B), (E), (H) baseline transmission rate, and (C), (F), (I) high transmission rate. The case counts of the unvaccinated individuals are depicted by the red line and the unit labels on the left-side y-axis, whereas the infected, vaccinated (breakthrough) cases are depicted by the green line and the unit labels on the right-side y-axis.

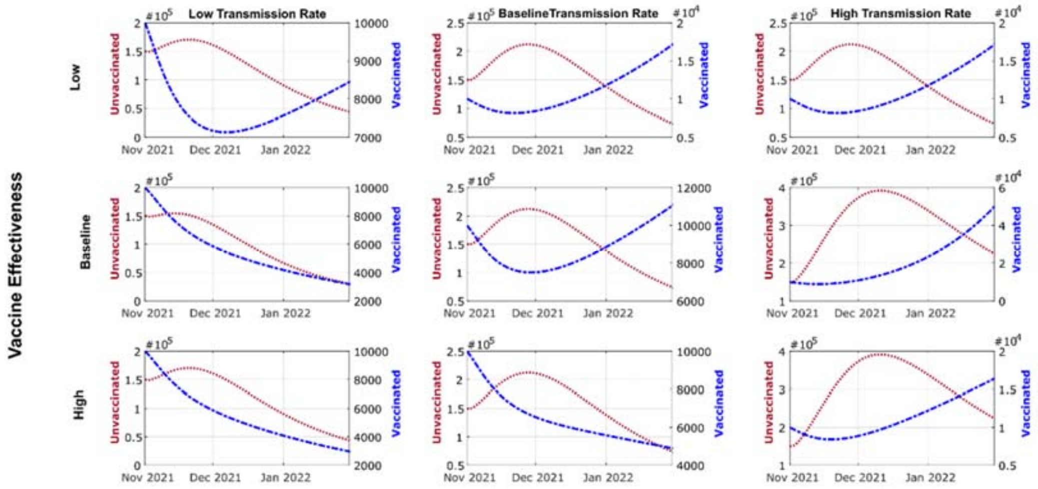

Figure. S5. Modelled projections of baseline vaccinated rate of symptomatic infections in unvaccinated and vaccinated subpopulations under different transmission rates and vaccine effectiveness. The low and high vaccination effectiveness denotes the lower bound and the higher bound of the 95% confidence intervals of the real-world data, respectively. Panels (A), (D), (G) represents scenarios with a low transmission rate, (B), (E), (H) baseline transmission rate, and (C), (F), (I) high transmission rate. The case counts of the unvaccinated individuals are depicted by the red line and the unit labels on the left-side y-axis, whereas the infected, vaccinated (breakthrough) cases are depicted by the green line and the unit labels on the right-side y-axis.

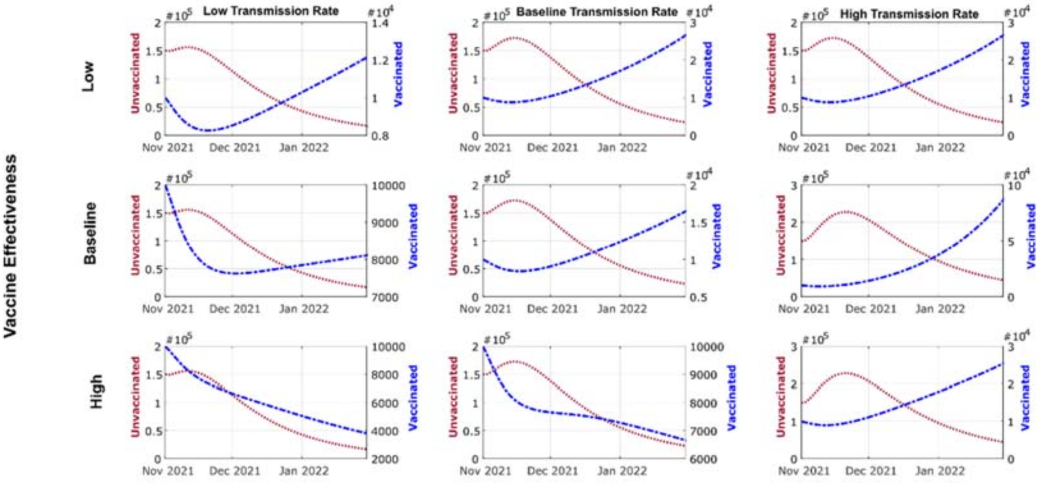

Figure. S6. Modelled projections of high vaccinated rate of symptomatic infections in unvaccinated and vaccinated subpopulations under different transmission rates and vaccine effectiveness. The low and high vaccination effectiveness denotes the lower bound and the higher bound of the 95% confidence intervals of the real-world data, respectively. Panels (A), (D), (G) represents scenarios with a low transmission rate, (B), (E), (H) baseline transmission rate, and (C), (F), (I) high transmission rate. The case counts of the unvaccinated individuals are depicted by the red line and the unit labels on the left-side y-axis, whereas the infected, vaccinated (breakthrough) cases are depicted by the green line and the unit labels on the right-side y-axis.

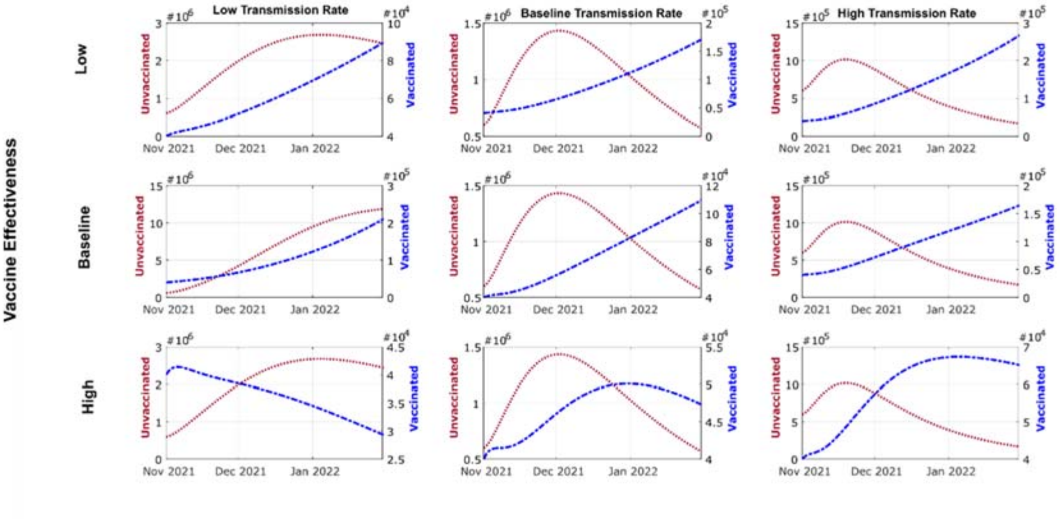

Figure. S7. Modelled projections of asymptomatic infections in unvaccinated and vaccinated subpopulations under different vaccination rates and vaccine effectiveness. The low and high vaccination effectiveness denotes the lower bound and the higher bound of the 95% confidence intervals of the real-world data, respectively. Panels (A), (D), (G) represents scenarios with a low (50%) vaccination rate, (B), (E), (H) baseline vaccination rate, and (C), (F), (I) high (200%) vaccination rate. The case counts of the unvaccinated individuals are depicted by the red line and the unit labels on the left-side y-axis, whereas the infected, vaccinated (breakthrough) cases are depicted by the green line and the unit labels on the right-side y-axis.

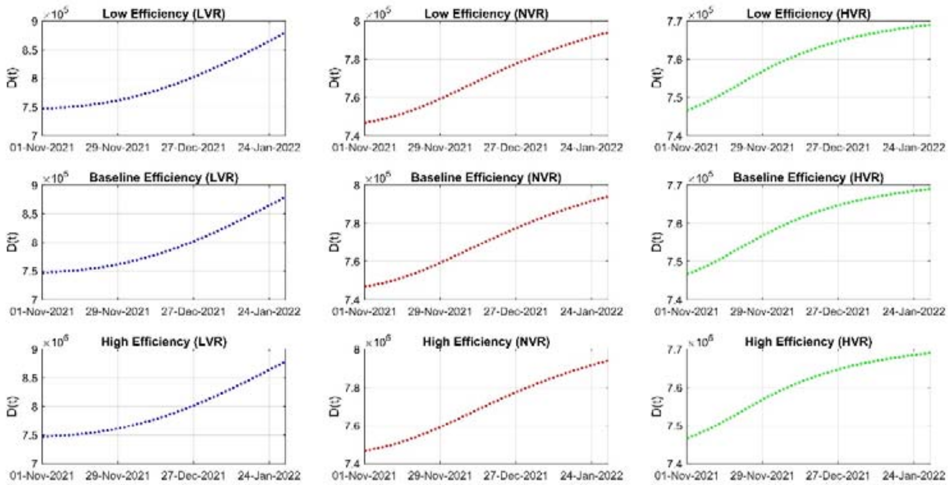

Figure. S8. Modelled projections of cumulative death in the US population under different vaccination rates and vaccine effectiveness. The low and high vaccination effectiveness denotes the lower bound and the higher bound of the 95% confidence intervals of the real-world data, respectively. Panels (A), (D), (G) represents scenarios with a low (50%) vaccination rate, (B), (E), (H) baseline vaccination rate, and (C), (F), (I) high (200%) vaccination rate. The case counts of the unvaccinated individuals are depicted by the red line and the unit labels on the left-side y-axis, whereas the infected, vaccinated (breakthrough) cases are depicted by the green line and the unit labels on the right-side y-axis.

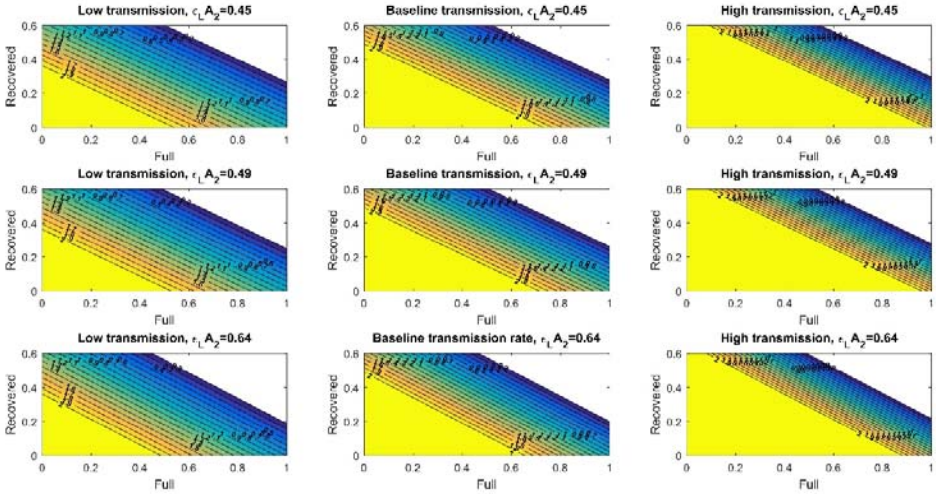

Figure. S9. Reproduction Control Number of the delta variant considering full immunity and recovered individuals for asymptomatic infections. The assumed vaccine effectiveness ($\boldsymbol{\varepsilon_{LA2}}$) parameter under each scenario is denoted on top of each panel and are inferred based on real-world data of individuals receiving the BNT162b2 vaccine. The heatmaps in left row consider a low transmission rate; the heatmaps in the center row panels consider a baseline transmission rate; and the heatmaps in the right row panels consider a high transmission rate.

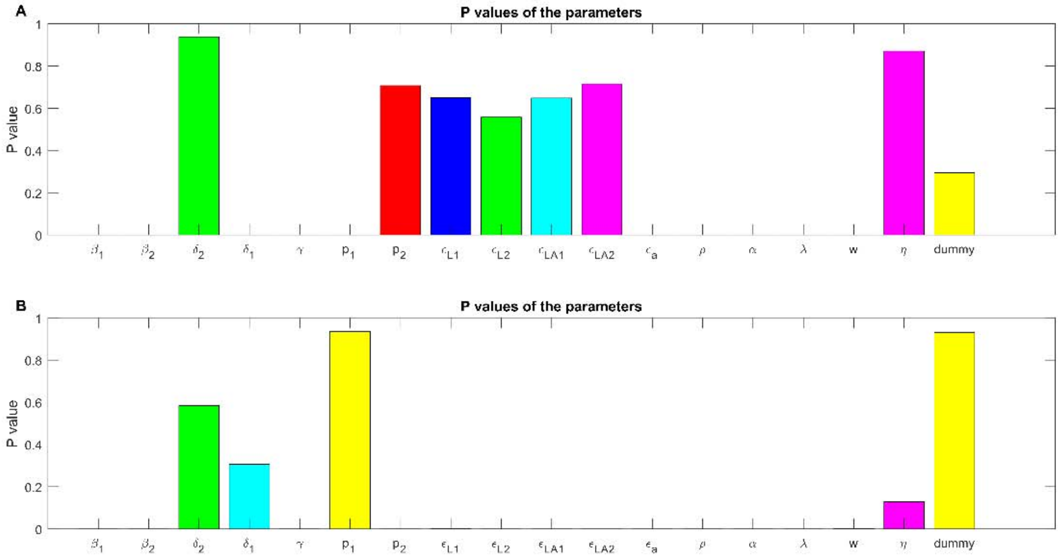

Figure. S10. Significance levels of the Partial Rank Correlation Coefficient (PRCC, depicted in Fig 6) for each parameter from the global sensitivity analyses based on our model, including the results for the (A) unvaccinated, and (B) vaccinated infected, symptomatic individuals. Visible bars where $P > 0.05$ denotes that the parameter was not significantly correlated with the said sub-population in the mathematical model.

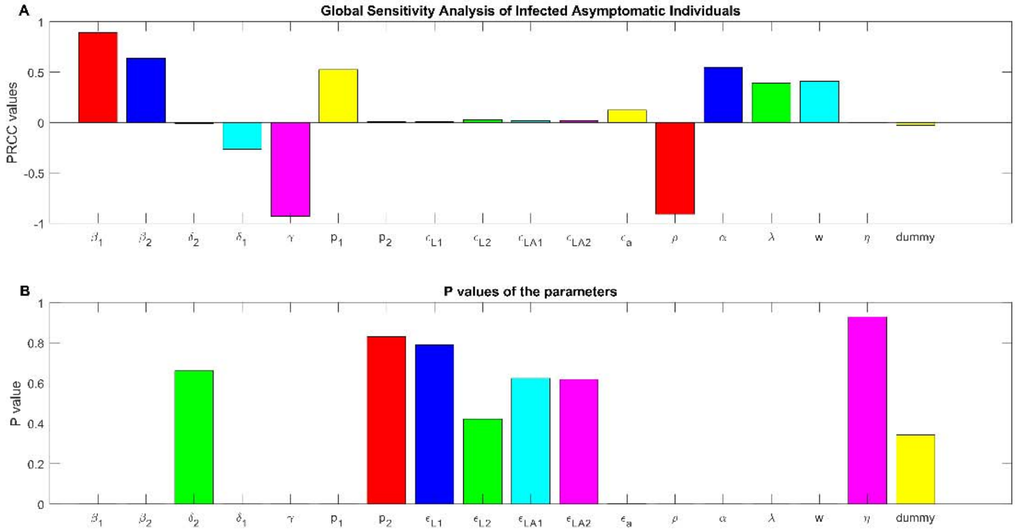

Figure. S11. Global sensitivity analyses of the parameters correlated with the unvaccinated, infected asymptomatic individuals in the mathematical model, including the (A) PRCC and (B) P value of each parameter. In the top panel, a positive PRCC indicates an increase in the parameters is correlated with an increase of the said sub-population, and a negative PRCC vice versa. In the bottom panel, parameters showing visible bars where P > 0.05 denotes that it was not significantly correlated with the said sub-population in the mathematical model.

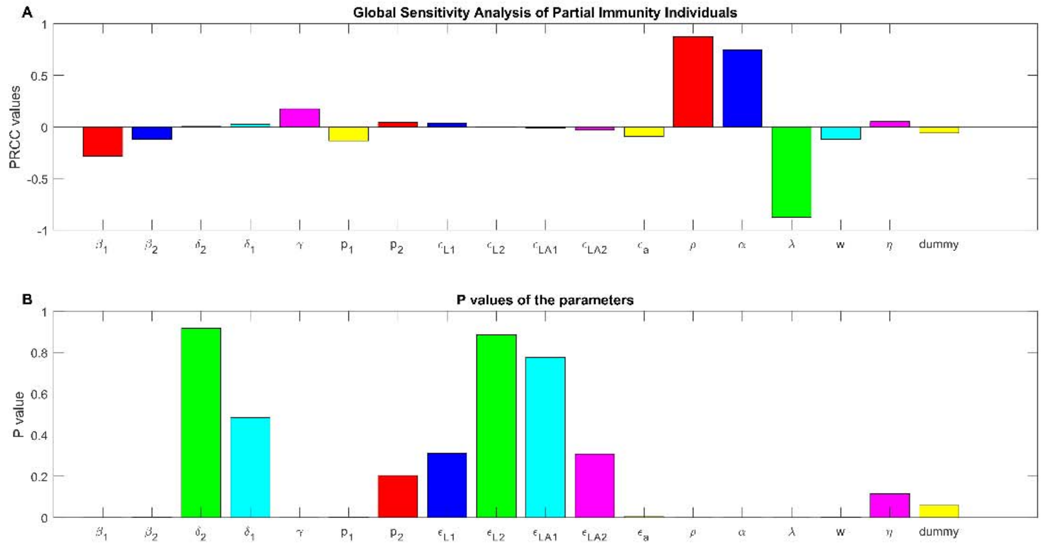

Figure. S12. Global sensitivity analyses of the parameters correlated with the partially vaccinated individuals (one dose) in the mathematical model, including the (A) PRCC and (B) P value of each parameter. In the top panel, a positive PRCC indicates an increase in the parameters is correlated with an increase of the said sub-population, and a negative PRCC vice versa. In the bottom panel, parameters showing visible bars where P > 0.05 denotes that it was not significantly correlated with the said sub-population in the mathematical model.

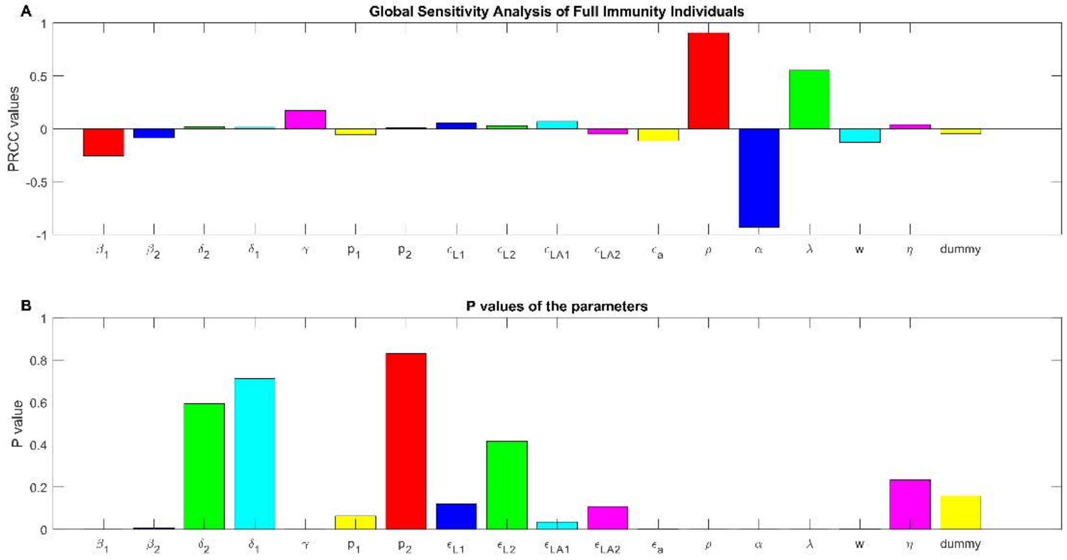

Figure. S13. Global sensitivity analyses of the parameters correlated with the fully vaccinated individuals (two doses) in the mathematical model, including the (A) PRCC and (B) P value of each parameter. In the top panel, a positive PRCC indicates an increase in the parameters is correlated with an increase of the said sub-population, and a negative PRCC vice versa. In the bottom panel, parameters showing visible bars where P > 0.05 denotes that it was not significantly correlated with the said sub-population in the mathematical model.

Table S1. Parameters used in the simulation of our mathematical model. Some parameters were fitted from data and the other were directly retrieved from the literature, including real-world studies and clinical trials.

Parameter	Meaning	Value	Goodness of fit of 95% Confidence Interval	Source
ε_a	All or nothing of the vaccine	0.0862	(0.0765-0.0959)	2
ρ	Vaccination rate	Variable		Fitted from data
β_1	Symptomatic infection rate	0.557	(0.4456-0.6684)	Fitted from data
β_2	Asymptomatic infection rate	0.08	(0.076-0.114)	Fitted from data
α	Waning rate of the vaccine	0.05555 or $1/180$ $(6\ months)$	(0.011-0.0037)	Approximated (1)
ε_{L1}	Vaccine effectiveness for one dose against symptomatic infection	0.35	(0.22-0.4)	2
ε_{L2}	Vaccine effectiveness for two doses for symptomatic infection	0.59	(0.5-0.78)	2
ε_{LA1}	Vaccine effectiveness for one dose against asymptomatic infection	0.30	(0.22-0.64)	2
ε_{LA2}	Vaccine effectiveness for two doses against asymptomatic infection	0.49	(0.45-0.64)	2
w	Latent period for individuals infected with delta variant	0.25 or $1/4$	Fixed	3
γ	Recovery rate of individuals	0.037	(0.0067-0.0673)	Fitted from data

δ_1	Death rate from unvaccinated individuals	0.0019	$(0.00152 - 0.00228)$	Fitted from data
δ_2	Death rate from vaccinated individuals	$1.9456 * 10^{-5}$	$(1.5564 * 10^{-5}, 2.3347 * 10^{-5})$	Approximated (4)
p_1	Proportion of symptomatic individuals of delta variant	0.12	Fixed	Approximated (7)
p_2	Proportion of symptomatic vaccinated individuals.	0.09	Fixed	Approximated (2)
η	Recovered individuals that get infected with the delta variant	0.0027	Fixed	Approximated (5-6)
λ	Time between first and second dose	0.0476	Fixed	7
μ_1	Relative transmission of one-dose vaccinated individuals	0.94	(0.84-1.02)	8
μ_2	Relative transmission of a two-dose vaccinated individuals	0.73	(0.59-0.9)	8

Table S2. Average value of the vaccination rate in the ranges of dates where vaccine rollout was carried in the US.

Date ranges	Average value of $\rho(t)$
**13th December-31st December**	0.0044331
**January 1st-January 31st**	0.008557
**February 1st -February 28th**	0.10235714
**1st of March-31st of March**	0.123516
**1st of April-30th of April**	0.10308
**1st of May- 31st of May**	0.1421
**1st of June-30th of June**	0.1519
**1st of July – 31st of July**	0.0523
**1st of August – 31th of August**	0.1241
**1st of September-30th of September**	0.1119
**1st of October-31st of October**	0.1613
**> of 1st of November**	0.05

References

1. Tartof, S.Y.; Slezak, J.M.; Fischer, H.; Hong, V.; Ackerson, B.K.; Ranasinghe, O.N.; Frankland, T.B.; Ogun, O.A.; Zamparo, J.M.; Gray, S.; et al. Effectiveness of mRNA BNT162b2 COVID-19 Vaccine up to 6 Months in a Large Integrated Health System in the USA: A Retrospective Cohort Study. *Lancet* **2021**, *398*, 1407–1416.
2. Elliott, P.; Haw, D.; Wang, H.; Eales, O.; Walters, C.; Ainslie, K.; Atchison, C.; Fronterre, C.; Diggle, P.; Page, A.; et al. REACT-1 Round 13 Final Report: Exponential Growth, High Prevalence of SARS-CoV-2 and Vaccine Effectiveness Associated with Delta Variant in England during May to July 2021. **2021**.
3. Sjödin, H.; Wilder-Smith, A.; Osman, S.; Farooq, Z.; Rocklöv, J. Only Strict Quarantine Measures Can Curb the Coronavirus Disease (COVID-19) Outbreak in Italy, 2020. *Euro Surveill.* **2020**, *25*, doi:10.2807/1560-7917.ES.2020.25.13.2000280.
4. Puranik, A.; Lenehan, P.J.; Silvert, E.; Niesen, M.J.M.; Corchado-Garcia, J.; O'Horo, J.C.; Virk, A.; Swift, M.D.; Halamka, J.; Badley, A.D.; et al. Comparison of Two Highly-Effective mRNA Vaccines for COVID-19 during Periods of Alpha and Delta Variant Prevalence. *medRxiv* **2021**, doi:10.1101/2021.08.06.21261707.
5. Gazit, S.; Shlezinger, R.; Perez, G.; Lotan, R.; Peretz, A.; Ben-Tov, A.; Cohen, D.; Muhsen, K.; Chodick, G.; Patalon, T. Comparing SARS-CoV-2 Natural Immunity to Vaccine-Induced Immunity: Reinfections versus Breakthrough Infections. *bioRxiv* 2021.
6. Kojima, N.; Klausner, J.D. Protective Immunity after Recovery from SARS-CoV-2 Infection. *Lancet Infect. Dis.* **2021**, doi:10.1016/S1473-3099(21)00676-9.
7. The Pfizer BioNTech (BNT162b2) COVID-19 Vaccine: What You Need to Know Available online: https://www.who.int/news-room/feature-stories/detail/who-can-take-the-pfizer-biontech-covid-19--vaccine (accessed on 17 September 2021).
8. Allen, H.; Vusirikala, A.; Flannagan, J.; Twohig, K.A.; Zaidi, A.; Chudasama, D.; Lamagni, T.; COVID-19 Genomics UK (COG-UK Consortium) Household Transmission of COVID-19 Cases Associated with SARS-CoV-2 Delta Variant (B.1.617.2): National Case-Control Study. *Lancet Reg Health Eur* **2021**, 100252.
